# Beyond pathogens: a narrative review of the immunological nexus of damage-associated molecular patterns and inflammasome activation in sterile AECOPD

**DOI:** 10.3389/fimmu.2026.1803770

**Published:** 2026-04-28

**Authors:** Chenwen Peng, Jingyan Wei, Fengao Liang, Jianfeng Chen, Lei Wu, Ying Huang, Zhenyan Huang, Chuanjian Lu

**Affiliations:** 1 Zhongshan Hospital of Traditional Chinese Medicine Affiliated to Guangzhou University of Traditional Chinese Medicine, Zhongshan, China; 2 Zhongshan Hospital of Traditional Chinese Medicine, Zhongshan, China; 3 Guangdong Provincial Hospital of Chinese Medicine, Guangdong Provincial Academy of Chinese Medical Sciences, Guangzhou, China

**Keywords:** AECOPD, damage-associated molecular patterns, DAMPs, immune regulatory mechanisms, NLRP3 inflammasome

## Abstract

The acute exacerbation of chronic obstructive pulmonary disease (AECOPD) has traditionally been attributed to pathogen-associated molecular patterns (PAMPs); however, emerging evidence highlights the critical role of damage-associated molecular patterns (DAMPs) in driving sterile inflammation during these episodes. The NLR Family Pyrin Domain Containing 3(NLRP3) inflammasome, a pivotal component of the innate immune system, mediates inflammatory responses that contribute significantly to the pathogenesis of AECOPD. Building upon the integration of current research on the molecular mechanisms of DAMP-NLRP3 inflammasome interaction, this review further emphasizes its immunological significance in sterile AECOPD. Furthermore, we explore how viral infections, such as the influenza virus, potentiate inflammasome activation and exacerbate inflammatory cascades, thereby influencing clinical outcomes. By integrating recent experimental and clinical findings, this article aims to provide a comprehensive understanding of the immune regulatory mechanisms involved in AECOPD beyond microbial triggers. Such insights are essential to advancing precision therapeutic strategies targeting inflammasome pathways and improving patient management.

## 1 Introduction

Chronic obstructive pulmonary disease (COPD) manifests primarily as persistent airflow limitation and chronic airway inflammation—a progressive condition that encompasses both obstructive bronchiolitis and emphysema. The disease is primarily caused by long-term exposure to noxious particles or gases, with cigarette smoking being the most significant risk factor. The pathophysiology of COPD is complex and multifactorial, involving chronic inflammation, airway remodeling, and destruction of lung parenchyma, which ultimately leads to exacerbations that can severely affect lung function and overall health (1).

Acute exacerbations of COPD (AECOPD) are defined as a sustained worsening of respiratory symptoms, primarily characterized by increased dyspnea, cough, and sputum production. These episodes reflect heightened airway inflammation and accelerated decline in lung function, significantly contributing to patient morbidity. The clinical diagnosis of AECOPD relies on comprehensive symptom assessment, complemented by objective diagnostic tools. Spirometry is essential for confirming the underlying diagnosis of COPD, with a post-bronchodilator FEV^1^/FVC ratio < 0.70 serving as the diagnostic hallmark. During acute exacerbations, lung function testing helps quantify the severity of airflow limitation and establish a baseline for the patient’s impairment, although performing high-quality spirometry in acute settings is challenging (2). Computed tomography (CT) scans are not used to diagnose AECOPD per se but play an indispensable role in differential diagnosis, helping to exclude alternative conditions that may mimic AECOPD—such as pulmonary embolism, pneumonia, or congestive heart failure. Notably, CT imaging during AECOPD frequently reveals acute-phase lung involvement, including pulmonary infiltrates, ground-glass opacities, and segmental consolidations, which are observed in a substantial proportion of patients. Studies have reported that approximately 60% of AECOPD patients present with lung infiltration on CT, with the extent of parenchymal infiltration correlating with inflammatory biomarkers such as neutrophil count (3). CT imaging also reveals structural abnormalities, including emphysema subtypes, bronchial wall thickening, and air trapping, which provide valuable prognostic information and guide long-term management strategies (4).

AECOPD represents a major driver of healthcare resource utilization worldwide. In addition to respiratory symptoms, these acute episodes are often accompanied by systemic effects, including fatigue and anxiety, leading to substantial healthcare resource use. Epidemiological data indicate that between 2010 and 2018, there were an estimated 7.5 million emergency department visits for AECOPD in the United States, accounting for approximately 14 per 1,000 adult ED visits (5). A Korean nationwide cohort study reported that patients with COPD experienced a mean of 0.2 severe AECOPD events per year and approximately 1.1 moderate or severe AECOPD events annually (6). A recent U.S. study further found that among patients discharged following hospitalization for AECOPD, 16.7% experienced COPD-related readmission within one year, with a readmission rate of 20.4 per 100 patient-years(10), and the one-year post-discharge mortality rate reached 18.2% (7). In Europe, an Italian study showed that among COPD patients requiring hospitalization for severe acute exacerbations, the one-year mortality rate was 25.6%, and the readmission rate was 20.1% (8). These data fully reveal the substantial clinical and economic burden of AECOPD, highlighting the urgency of improving preventive and therapeutic strategies.

**Figure 1:**
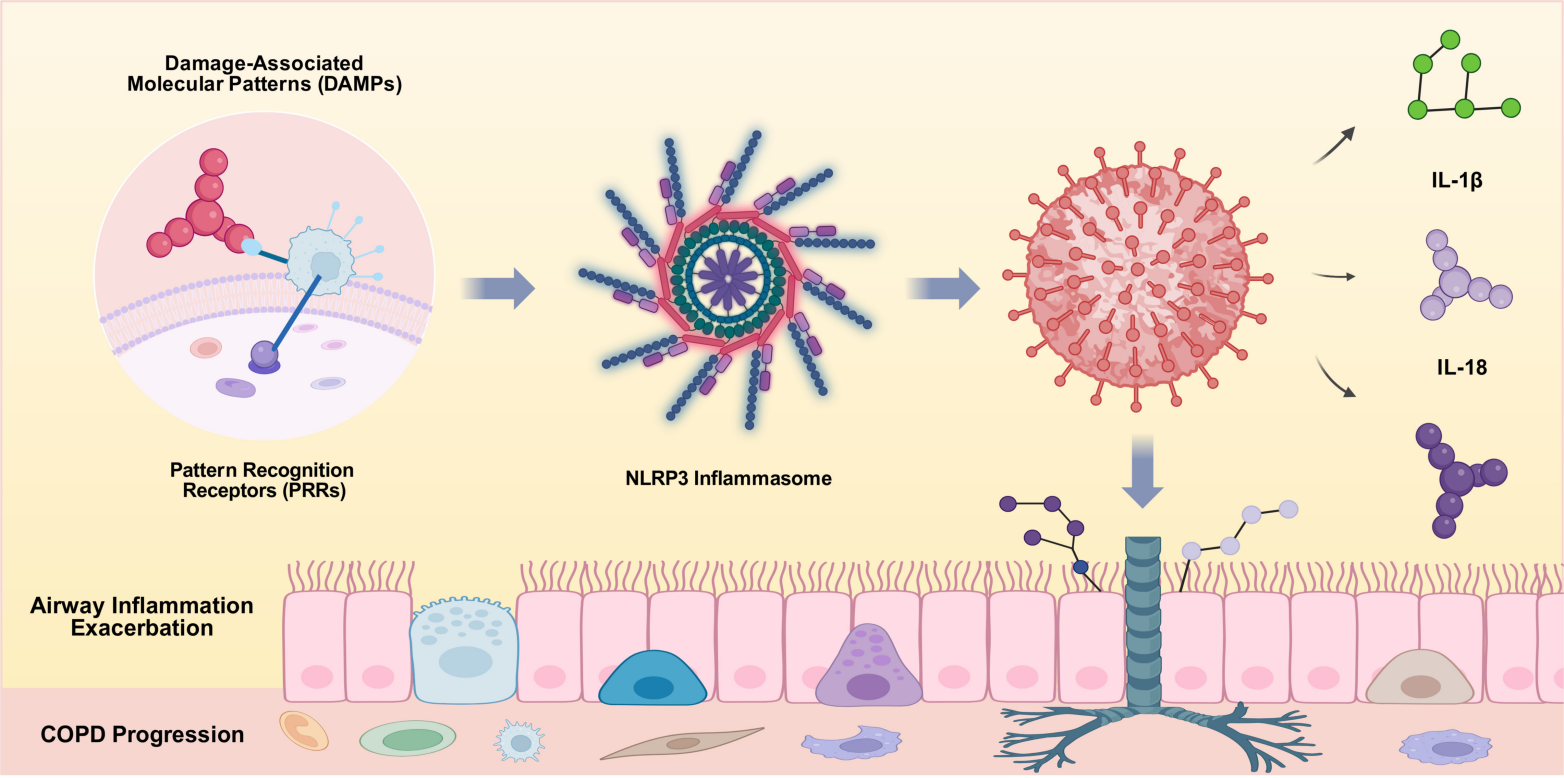
DAMPs interact with PRRs on immune cells to activate the NLRP3 inflammasome, leading to the release of pro-inflammatory cytokines and exacerbation of airway inflammation.

Traditionally, AECOPD has been attributed primarily to respiratory infections, particularly those caused by bacteria and viruses. However, it is noteworthy that a considerable proportion of exacerbation cases show no detectable pathogens. Epidemiological studies provide supporting data for this observation: a Korean study involving 736 patients reported that 36.7% of AECOPD cases were pathogen-negative (9); another Korean multicenter study with 1,186 AECOPD patients found a pathogen-negative rate of approximately 44.5% (10); and a UK study of 264 cases reported a pathogen-negative rate of 49% among AECOPD patients (11). Collectively, these data suggest that an estimated 35–50% of AECOPD cases may be driven by non-infectious factors. This finding has prompted researchers to reconsider the role of non-infectious factors in the pathogenesis of acute exacerbations. Among these, damage-associated molecular patterns (DAMPs) have emerged as critical players in the inflammatory processes associated with COPD exacerbations. DAMPs are endogenous molecules released from damaged or dying cells that can trigger innate immune responses by interacting with pattern recognition receptors (PRRs) on immune cells. This interaction can lead to the activation of inflammasomes, particularly the NLRP3 inflammasome, which plays a significant role in the release of pro-inflammatory cytokines such as interleukin-1β (IL-1β) and interleukin-18(IL-18), further perpetuating the inflammatory cycle (12), as shown in **Figure 1**.

**Figure 2:**
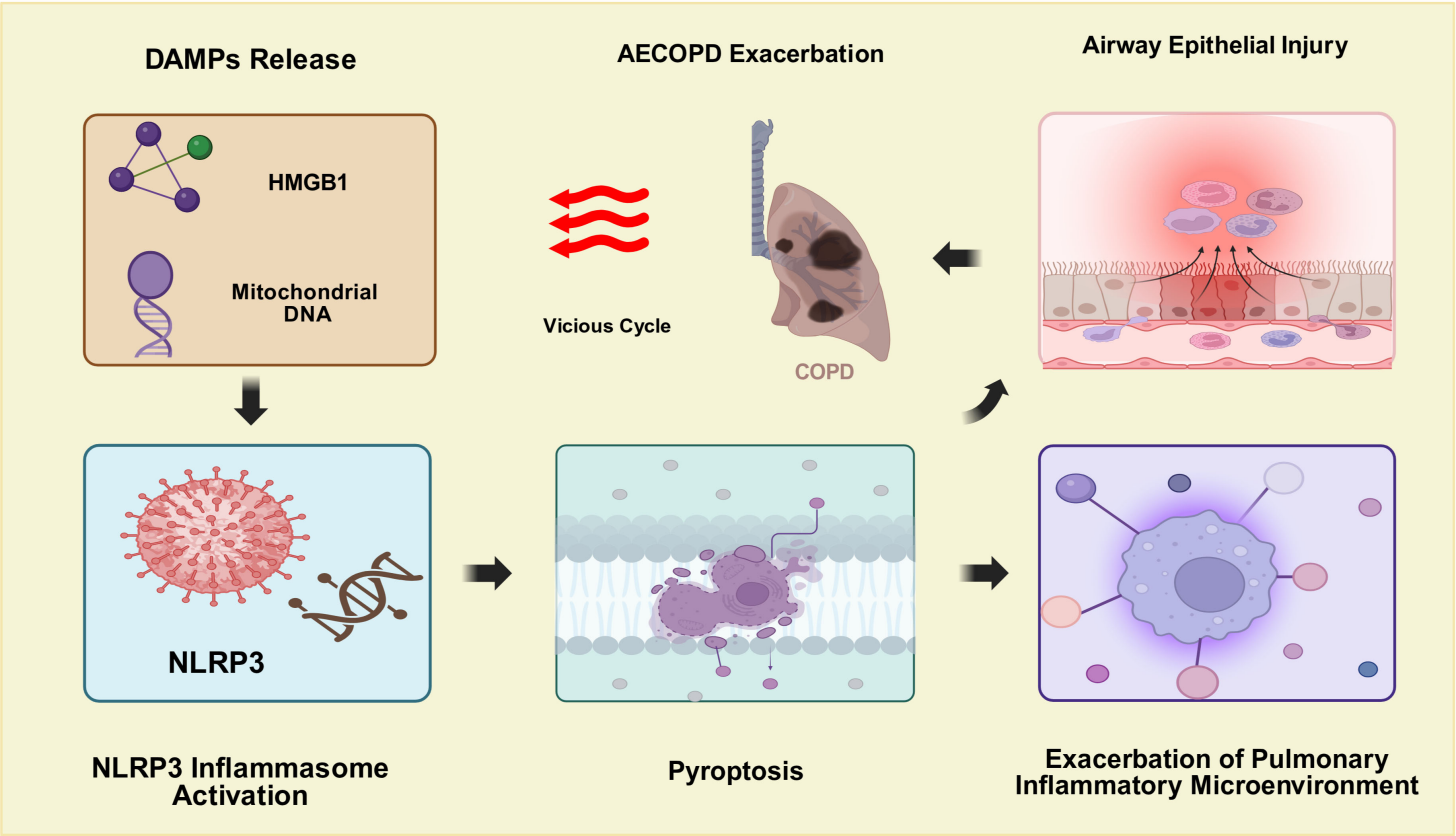
Schematic diagram of the vicious cycle of AECOPD exacerbation driven by sterile inflammation. This figure illustrates that following airway epithelial injury caused by AECOPD, damaged cells release DAMPs such as HMGB1 and mitochondrial DNA, which activate the NLRP3 inflammasome and induce pyroptosis. This process exacerbates the pulmonary inflammatory microenvironment, further impairs the airway epithelium, and ultimately forms a vicious cycle that drives AECOPD progression.

The NLR Family Pyrin Domain Containing 3(NLRP3) inflammasome is a key component of the innate immune system, acting as a sensor for various DAMPs and PAMPs (pathogen-associated molecular patterns). Its activation leads to a cascade of inflammatory responses that can exacerbate airway inflammation and contribute to the progression of COPD. In the context of sterile inflammation, such as that seen in COPD exacerbations not directly caused by infection, DAMPs released from damaged airway epithelial cells can activate the NLRP3 inflammasome, resulting in the secretion of inflammatory mediators that exacerbate the underlying airway inflammation (13, 14). Beyond cytokine release, studies in animal models and cell lines have shown that specific DAMPs such as high-mobility group box 1 (HMGB1) and mitochondrial DNA can drive NLRP3-mediated pyroptosis, an inflammatory form of programmed cell death. This process not only contributes to the inflammatory milieu in the lungs but may also lead to further damage and dysfunction of the airway epithelium, creating a vicious cycle that perpetuates exacerbations and accelerates disease progression (15), as shown in **Figure 2**. However, it is important to acknowledge that the current understanding of the DAMP-NLRP3 inflammasome axis in AECOPD is largely derived from animal models and *in vitro* cellular experiments. While these studies have provided important theoretical foundations for understanding disease mechanisms, direct clinical evidence remains relatively limited. Throughout this review, we endeavor to clearly distinguish the source of evidence—findings from animal models or cell-based experiments are indicated as such, whereas data obtained from human COPD studies are explicitly noted. This distinction is critical for evaluating the translational potential of these mechanistic insights.

This review synthesizes current evidence to demonstrate that sterile, DAMP-driven inflammation centered on NLRP3 inflammasome activation constitutes a key pathogenic axis in AECOPD. This mechanism can operate independently of infection and is substantially amplified by viral and other stimuli, thereby providing a unified pathophysiological explanation for exacerbations triggered by diverse factors. Future research should focus on exploring how to modulate this signaling axis to develop more effective therapeutic strategies. This offers a new theoretical foundation and potential direction for ultimately improving the clinical management and disease prognosis of COPD exacerbations. However, translating insights from this pathogenic model into clinical advances requires a critical appraisal of unresolved controversies and methodological challenges within the field. Key questions persist, such as defining the hierarchical contributions of specific DAMPs, including HMGB1, S100 calcium-binding protein A8/A9 (S100A8/A9), and mitochondrial DNA (mtDNA), across the COPD stability-exacerbation continuum (16). Additionally, elucidating how signals from diverse DAMPs are integrated to precisely activate the NLRP3 inflammasome in the airway microenvironment remains critical. Furthermore, while the NLRP3 inflammasome is a compelling drug target, therapeutic strategies face unique challenges in AECOPD, including the risk of immunosuppression, the presence of MCC950-resistant NLRP3 variants, and the need to differentiate its role from alternative inflammasomes in sterile inflammation (17, 18). This review will synthesize the current evidence and provide a comparative analysis of these conflicting insights and limitations, aiming to identify crucial gaps for future research focused on mechanistic validation and therapeutic translation.

## 2 Methods

### 2.1 Search strategy

This narrative review was conducted following the PRISMA (Preferred Reporting Items for Systematic Reviews and Meta-Analyses) guidelines to ensure transparency. We systematically searched PubMed, Web of Science, and Scopus for literature published from January 2020 to the present. The search strategy combined MeSH terms and free-text keywords across three core domains using Boolean operators. The first domain, Disease Context, included terms such as “Chronic Obstructive Pulmonary Disease (COPD)”, “Acute Exacerbation of COPD (AECOPD)”, “Sterile exacerbation”, “Non-infectious exacerbation”, and “Virus-induced exacerbation”. The second domain, DAMPs and Sterile Triggers, encompassed “Damage-Associated Molecular Patterns (DAMPs)”, “Alarmins”, “Heat Shock Protein 70 (Hsp70, eHsp70)”, “Extracellular ATP (Adenosine Triphosphate)”, “HMGB1 (High Mobility Group Box 1)”, “S100A8”, “S100A9”, “Calprotectin”, “Uric Acid”, “Monosodium Urate”, “Cell-free DNA”, “Mitochondrial DNA (mtDNA)”, and “RAGE ligands”. The third domain, Inflammasome and Signaling, covered “Inflammasome”, “NLRP3 (NOD-like receptor family pyrin domain containing 3)”, “AIM2”, “NLRC4”, “Caspase-1”, “Interleukin-1β (IL-1β)”, “Interleukin-18 (IL-18)”, “Pyroptosis”, “Gasdermin D (GSDMD)”, “P2X7 receptor”, “P2X4 receptor”, “TLR4”, “RAGE”, “Potassium efflux”, and “K+ efflux”. Terms within each domain were connected using the Boolean operator OR, while the three domains were combined using the Boolean operator AND to maximize sensitivity and relevance. Additionally, reference lists of key articles and recent reviews were manually screened to identify relevant studies missed by the database search.

### 2.2 Selection criteria

Inclusion criteria were: (i) original research, including *in vitro*, *in vivo*, or clinical studies, focusing on DAMPs or sterile inflammasome activation in COPD/AECOPD; (ii) articles published in English; and (iii) studies providing clear mechanistic insights or clinical data on sterile exacerbations. Exclusion criteria included: (i) studies that focused exclusively on infectious AECOPD without addressing sterile mechanisms or DAMP-mediated pathways; (ii) types of non-original research, such as editorials and conference abstracts; and (iii) articles lacking full-text availability or methodological clarity.

### 2.3 Screening results

**Figure 3:**
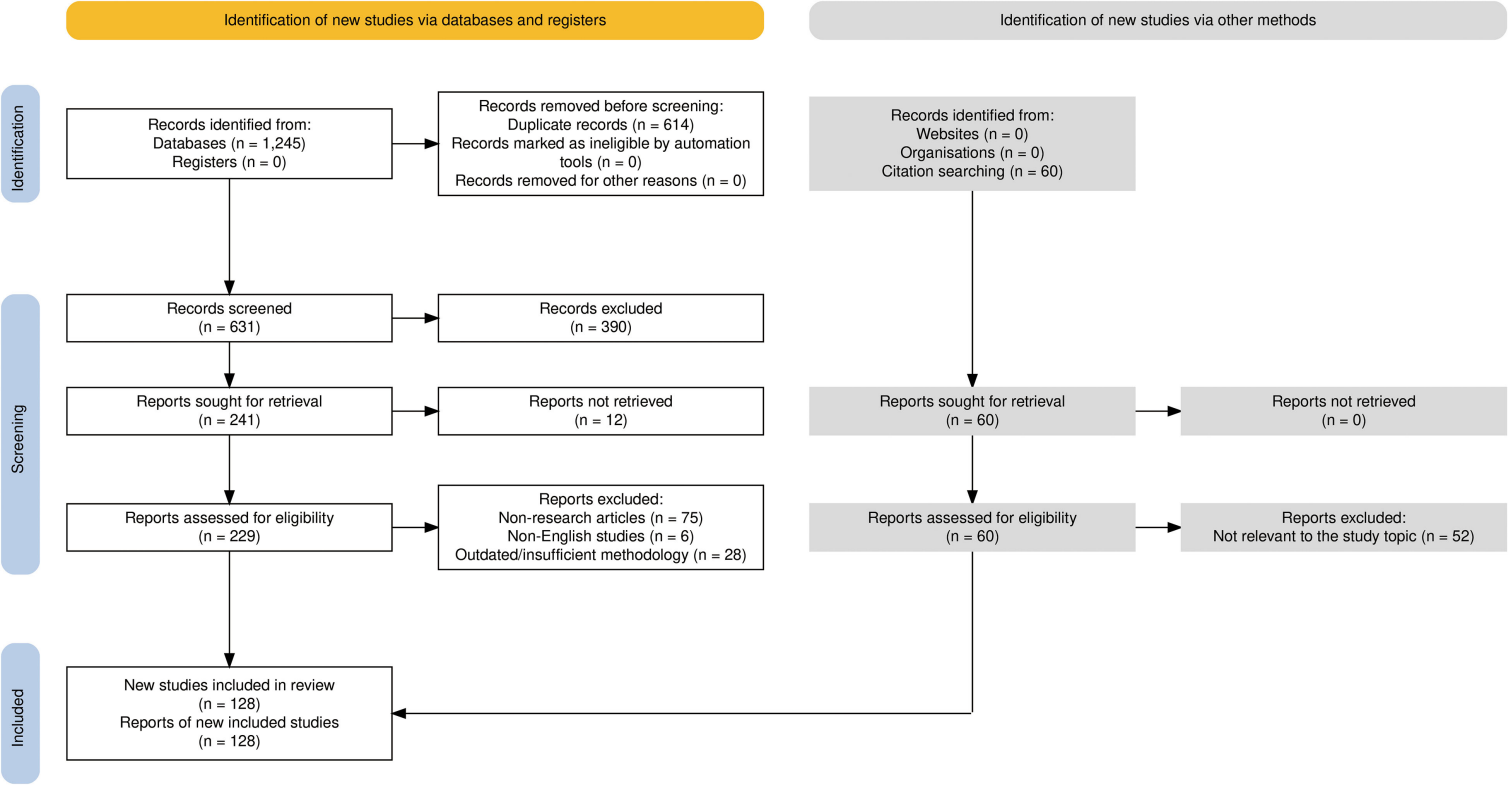
PRISMA flowchart for literature search.

This study followed the PRISMA framework for literature screening (19)(**Figure 3**). Two authors independently conducted database searches across PubMed, Web of Science, and Scopus using the described strategy. The initial combined search retrieved 1,245 records. After removing 614 duplicates using an automated reference manager (EndNote 21), 631 unique records remained for initial screening. The titles and abstracts of these records were screened against the selection criteria. This process led to the exclusion of 390 articles that were unrelated to the research topic. The remaining 241 articles proceeded to full-text retrieval. Of these, 12 articles were inaccessible and were excluded. A detailed eligibility assessment was then carried out on the remaining 229 articles. This led to the removal of 75 non-research articles, 6 non-English studies, and 28 studies excluded due to outdated findings or insufficient methodological relevance to the review’s focus on sterile inflammation in AECOPD. Subsequently, citation tracking from the reference lists of key included articles and recent major reviews was conducted. This process identified 60 potentially relevant articles not captured by the initial database search. After evaluation, 52 of these were excluded as they were published before 2020, were duplicates, or were unrelated to the core mechanistic focus of this review. The remaining 8 articles met the inclusion criteria and were included in the review. Ultimately, a total of 128 studies that met all inclusion criteria were included in this narrative review. The final body of literature provides a foundation for discussing sterile inflammation in AECOPD, encompassing both direct and highly supportive evidence.

### 2.4 Immunopathological basis of AECOPD

#### 2.4.1 Definition and clinical manifestations of AECOPD

AECOPD is defined as a sustained deterioration in respiratory symptoms, marked notably by increased dyspnea, cough, and sputum production. Such episodes typically reflect heightened airway inflammation and accelerated lung function decline, collectively contributing to significant impairment in patients’ health status and quality of life. Beyond respiratory manifestations, AECOPD frequently encompasses systemic effects, including fatigue and anxiety, and drives substantial healthcare utilization—accounting for millions of emergency visits and hospitalizations annually, thus representing a major burden on health systems (20).

Traditionally considered to result from bacterial or viral infections, AECOPD is now increasingly linked to non-infectious triggers. These “aseptic” exacerbations, constituting a growing clinical subset, may be initiated by environmental exposures (e.g., air pollution, allergens) or underlying comorbidities (21, 22). Such factors can provoke airway inflammation independently of detectable pathogens, underscoring the multifactorial etiology of AECOPD, in which infectious and sterile stimuli often converge to drive symptom escalation.

The clinical diagnosis of AECOPD relies heavily on the assessment of changes in symptoms, along with the evaluation of inflammatory biomarkers. The identification of exacerbations is often made through clinical judgment, which can vary among healthcare providers. Symptoms such as increased cough, sputum production, and breathlessness are critical indicators, but the assessment may also include laboratory tests to measure inflammatory markers like C-reactive protein (CRP) and the neutrophil-lymphocyte ratio (NLR), which clinical studies have shown to correlate with the severity of exacerbations (23). These biomarkers can provide additional insights into the inflammatory status of the patient and help guide treatment decisions. However, the utility of these biomarkers remains limited by their lack of specificity: CRP elevation occurs in both infectious and sterile inflammation, while NLR can be confounded by corticosteroid use or concurrent cardiovascular events. This underscores a critical gap—current diagnostic tools cannot reliably distinguish between mechanistically distinct exacerbation endotypes, hindering precision management.

Moreover, the complexity of AECOPD is underscored by the fact that many patients present with overlapping symptoms from other comorbidities, such as heart failure or pneumonia, which can complicate the clinical picture and delay appropriate management (24). Therefore, a comprehensive approach that considers the patient’s entire clinical context, including their history of exacerbations, comorbid conditions, and environmental exposures, is essential for accurate diagnosis and effective management.

#### 2.4.2 The role of the immune system in AECOPD

The innate immune system constitutes a central pathogenic driver in AECOPD, where dysregulated responses amplify tissue injury and disease progression. Upon exacerbation onset, pulmonary macrophages sense both microbial and endogenous damage-associated molecular patterns, initiating a pro-inflammatory cascade through cytokine and chemokine release (25). This signaling rapidly recruits neutrophils that intensify inflammation via oxidative burst and protease secretion, directly contributing to airway epithelial damage. Concurrently, activated epithelial cells not only perpetuate inflammation through autocrine and paracrine signaling but also promote structural remodeling of the airways (26). While this coordinated cellular response is physiologically protective, its chronic dysregulation establishes a self-sustaining inflammatory circuit that underlies the airway obstruction and parenchymal destruction characteristic of exacerbated COPD.

The cytokine and chemokine surge during AECOPD drives a self-amplifying inflammatory cascade that mediates airway injury and structural remodeling. Elevations in pivotal mediators, including interleukin-6 (IL-6), IL-1β, and tumor necrosis factor (TNF)-α, not only recruit and activate leukocytes, sustaining chronic inflammation, but also promote pathological changes such as mucus hypersecretion and subepithelial fibrosis. Notably, one study reported elevated levels of IL-6, IL-1β, and TNF-α in both stable and exacerbated COPD patients compared to controls, yet only CRP and IL-17A showed significant correlation with symptom severity and exacerbation frequency34435653. This suggests that while a chronic inflammatory background is pervasive, specific mediators—particularly IL-17A—may serve as more dynamic indicators of acute worsening, highlighting the need to differentiate baseline inflammation from exacerbation-specific signals. This persistent inflammatory milieu exacerbates airflow limitation and contributes to the development of systemic comorbidities, including cardiovascular disease, thereby complicating clinical management. Furthermore, inflammation-induced airway hyperreactivity and impaired local defense increase susceptibility to recurrent infections, establishing a vicious cycle that perpetuates disease progression (27). Immune dysregulation represents another key factor influencing both the progression of COPD and its acute exacerbations. This dysregulated state typically manifests as elevated systemic inflammatory markers, such as CRP and fibrinogen. Concurrently, impaired immune function may diminish the efficacy of commonly used interventions, including corticosteroids, thereby complicating the clinical management of acute exacerbations (28). Therefore, a deeper understanding of these immune mechanisms is essential for advancing targeted therapeutic strategies capable of effectively controlling exacerbations and improving clinical outcomes in patients (29).

#### 2.4.3 Differences and connections between PAMPs and DAMPs

PAMPs and DAMPs are critical components of the innate immune system, each playing distinct yet interconnected roles in the immune response. PAMPs are derived from exogenous pathogens, including bacterial lipopolysaccharides (LPS) and viral RNA, which are recognized by the immune system as indicators of infection. These molecular patterns are typically conserved structures found in a variety of microorganisms and serve as signals to initiate immune responses through PRRs such as Toll-like receptors (TLRs) (30). In contrast, DAMPs are endogenous molecules released by damaged or dying cells, reflecting the state of tissue injury or stress. DAMPs such as extracellular heat shock protein 70 (eHsp70) and adenosine triphosphate (ATP) can trigger immune activation independently of pathogens (31). This intrinsic function as endogenous danger signals underscores the role of DAMPs not only in initiating sterile inflammation but also in modulating tissue homeostasis and coordinating repair following cellular injury.

The activation of immune responses by both PAMPs and DAMPs occurs through PRRs, which recognize these molecular patterns and trigger downstream signaling cascades that lead to inflammation and immune activation. This shared mechanism underscores a complex interplay where PAMPs and DAMPs can modulate each other’s effects. For instance, the presence of DAMPs can enhance the immune response triggered by PAMPs, leading to a more robust inflammatory reaction (32). This synergy may explain why viral infections—which induce both PAMP release (viral RNA) and DAMP liberation (e.g., ATP from stressed epithelium)—often precipitate the most severe exacerbations (33). However, it also complicates efforts to attribute inflammation to a single trigger in real-world settings, where mixed insults are likely the norm rather than the exception. This synergistic effect can be particularly important in conditions such as sepsis, where both PAMPs and DAMPs are present, contributing to the severity of the immune response and influencing clinical outcomes. Furthermore, the simultaneous recognition of both PAMPs and DAMPs can lead to a more nuanced immune response, balancing the need to eliminate pathogens while also promoting tissue repair and preventing excessive inflammation (34).

Moreover, the relationship between PAMPs and DAMPs extends to their roles in various diseases, including chronic inflammatory conditions and cancer. In these contexts, the dysregulation of PAMP and DAMP signaling can contribute to pathogenesis, highlighting the importance of understanding their interactions (35). For example, in cancer, tumor-derived DAMPs can activate immune responses that promote tumor growth and metastasis, while also engaging PRRs that might otherwise target the tumor for destruction (36). This duality illustrates the complexity of immune responses where PAMPs and DAMPs not only serve as indicators of infection or injury but also play roles in disease progression.

#### 2.4.4 Structure and activation mechanism of the NLRP3 inflammasome

The NLRP3 inflammasome is a vital component of the innate immune system, acting as a multi-protein complex that orchestrates the inflammatory response upon sensing various danger signals. Structurally, the NLRP3 inflammasome comprises three essential components: the NLRP3 receptor, the adaptor protein apoptosis-associated speck-like protein containing a caspase recruitment domain(ASC), and the effector protein caspase-1 (37). The NLRP3 receptor is characterized by three distinct domains: the NACHT domain (named for its presence in NAIP, CIITA, HET-E, and TP1), the leucine-rich repeat (LRR) domain, and the pyrin domain (PYD). These domains play crucial roles in NLRP3 activation and function.

The assembly of the NLRP3 inflammasome begins with the recognition of DAMPs or PAMPs, leading to a two-signal activation process. Signal 1, often referred to as priming, involves the transcriptional upregulation of NLRP3 and pro-IL-1β through pathways such as NF-κB signaling. This initial signal prepares the NLRP3 receptor for subsequent activation (38). Signal 2, or activation, is triggered by various stimuli, including potassium efflux, mitochondrial dysfunction, and the presence of reactive oxygen species (ROS), culminating in the oligomerization of NLRP3 and the recruitment of ASC. This assembly forms a large, filamentous complex that facilitates the activation of caspase-1, which processes pro-inflammatory cytokines IL-1β and IL-18 into their active forms, leading to their release and the induction of pyroptosis, a form of inflammatory cell death (39).

The activation of the NLRP3 inflammasome is a highly regulated process, with various endogenous and exogenous signals capable of inducing its assembly. For instance, environmental irritants, metabolic dysregulation, and cellular stress can all serve as triggers. Recent studies have elucidated the structural dynamics of NLRP3, revealing that its inactive form exists as a double-ring structure that shields its PYD domains from the cytosol. Upon activation, this structure undergoes significant conformational changes, allowing the PYD domains to interact with ASC, leading to the formation of the inflammasome complex. The necessity of ATP and the centrosomal kinase NIMA-related kinase 7(NEK7) in this process has been highlighted, as these factors facilitate the transition from the inactive to the active state of NLRP3 (40, 41). Furthermore, mutations in NLRP3 that affect its oligomerization can lead to aberrant activation, contributing to autoinflammatory diseases.

The intricate interplay between NLRP3 and various cellular components underscores the complexity of its activation. For example, potassium efflux is a critical upstream event that promotes the conformational change necessary for NLRP3 activation. This efflux is often mediated by ion channels such as P2X purinoceptor 7 (P2X7) and pannexin-1 (PANX1), which facilitate the release of potassium ions from the cytoplasm (42). Additionally, mitochondrial factors, including mitochondrial DNA and ROS, have been implicated in the activation of NLRP3, highlighting the role of mitochondrial health in regulating inflammasome activity. Dysregulation of these processes can lead to chronic inflammation and has been associated with numerous diseases, including metabolic disorders and neurodegenerative conditions (42–44). The elucidation of structural and mechanistic principles underlying NLRP3 inflammasome activation provides critical insights into its physiological and pathophysiological functions, while simultaneously revealing novel therapeutic targets for inflammasome-driven disorders. Consequently, the design and development of selective small-molecule inhibitors capable of precisely disrupting NLRP3 assembly or signaling constitute a major focus of contemporary biomedical research. Advances in this domain hold significant promise for the treatment of a broad spectrum of inflammatory pathologies (45).

#### 2.4.5 Interaction between DAMPs and NLRP3 inflammasome in COPD: new insights

The DAMP-mediated activation of the NLRP3 inflammasome, particularly through signaling molecules such as eHsp70 and ATP, represents a key pathogenic pathway in COPD. As endogenous signals released under cellular stress, these DAMPs potently trigger NLRP3 inflammasome assembly, leading to enhanced production of pro-inflammatory cytokines, including IL-1β. *In vitro* experimental studies confirm that both eHsp70 and ATP upregulate NLRP3 and IL-1β expression in monocytes and bronchial epithelial cells, amplifying the inflammatory cascade during acute exacerbations (46). This inflammasome-centered mechanism is especially significant in driving and sustaining pulmonary inflammation in COPD, highlighting its role as a critical regulator of exacerbation severity and progression.

Simultaneously, it is increasingly recognized that DAMPs are not a monolithic group but may serve distinct roles within the spectrum of chronic inflammatory diseases such as COPD (47). Emerging evidence suggests a conceptual dichotomy: certain DAMPs, such as cell-free or mitochondrial DNA, primarily function as markers of chronic disease severity and mortality risk, reflecting ongoing, cumulative tissue damage and immune activation over time (48). In contrast, other DAMPs, particularly specific ligands for the receptor for advanced glycation end products (RAGE) such as the S100A8/A9 heterodimer (calprotectin), are released more robustly during acute cellular stress (49). These DAMPs act as potent acute exacerbation triggers by directly engaging pattern recognition receptors (e.g., TLR4, RAGE) and initiating the assembly of downstream signaling complexes, such as the NLRP3 inflammasome, thereby driving the fulminant sterile inflammation characteristic of an acute event (50, 51). This functional heterogeneity resolves an apparent paradox in the literature: while mtDNA levels correlate with long-term mortality, they show poor predictive value for imminent exacerbations; conversely, S100A8/A9 spikes acutely but does not track with baseline FEV^1^. Recognizing this distinction is therefore not merely academic—it informs biomarker selection for different clinical purposes (prognosis vs early warning).

Moreover, the response to DAMPs is not uniform across different cell types, which plays a significant role in determining the extent of inflammation in COPD. For example, monocytes and epithelial cells exhibit distinct responses to DAMPs, with variations in the expression of receptors such as TLRs and purinergic receptors that mediate the effects of eHsp70 and ATP (46). This differential response can influence the degree of inflammation and tissue damage, as seen *in vitro* studies where monocyte-derived macrophages and bronchial epithelial cells showed varying levels of NLRP3 activation and cytokine release when exposed to these DAMPs (52). Such findings underscore the complexity of the immune response in COPD, where the cellular context and the type of DAMPs present can significantly modulate the inflammatory response, potentially leading to either exacerbation or resolution of inflammation.

Recent evidence also suggests that DAMPs play a regulatory role in the context of sterile inflammation, particularly during non-infectious exacerbations of COPD. A clinical study has reported the presence of elevated levels of DAMPs such as eHsp70 and ATP in the peripheral blood of patients with end-stage COPD highlights their potential as biomarkers for disease exacerbation and progression (53). However, the clinical utility of circulating DAMPs remains uncertain. Elevated plasma levels may reflect systemic consequences of advanced disease—such as comorbid cardiovascular or renal injury—rather than active airway-specific inflammation. Without paired airway sampling, peripheral DAMP measurements should be interpreted as non-specific indicators of overall disease burden rather than precise triggers of exacerbation. Furthermore, targeting DAMPs and their signaling pathways may represent a novel therapeutic strategy for managing COPD. By modulating the activity of the NLRP3 inflammasome, it may be possible to attenuate the inflammatory responses that characterize acute exacerbations, thus improving patient outcomes. This therapeutic angle is supported by findings that interventions aimed at inhibiting NLRP3 activation can reduce inflammation and improve lung function in preclinical models of COPD (54). Collectively, the DAMP–NLRP3 axis not only advances our understanding of the immunological basis of COPD but also provides a translational framework for developing targeted therapies aimed at mitigating inflammatory pathology in this chronic respiratory disease (55).

#### 2.4.6 Distinguishing sterile from infectious AECOPD: a mechanistic and immunological comparison

Although the NLRP3 inflammasome serves as a central hub in the pathogenesis of AECOPD, the upstream signals that trigger its activation differ fundamentally depending on the nature of the inciting insult (28). Therefore, distinguishing between sterile and infectious AECOPD is not merely an academic exercise but a clinical necessity with profound therapeutic implications. **Table 1** provides a comprehensive comparison of the key features distinguishing these two exacerbation phenotypes. While both entities converge on NLRP3 inflammasome activation and subsequent IL-1β/IL-18 release, their initiating signals, upstream pathways, and broader immune contexts differ substantially (25). In infectious AECOPD, PAMPs derived from bacteria or viruses serve as the primary triggers, engaging canonical TLR signaling and often eliciting a robust adaptive immune response. Conversely, sterile AECOPD is driven by endogenous DAMPs—such as eHsp70, ATP, and HMGB1—released from stressed or injured cells, activating pattern recognition receptors including RAGE and P2X7 in the absence of microbial components (56).

**Table 1:** Key distinctions between sterile and infectious AECOPD.

Feature	Sterile AECOPD	Infectious AECOPD
Primary Triggers	Endogenous danger signals; Environmental irritants (cigarette smoke, air pollution, ozone); Allergens; Ischemia/reperfusion; Mechanical injury	Exogenous pathogens: Bacteria (*H. influenzae*, *S. pneumoniae*, *M. catarrhalis*); Viruses (Rhinovirus, Influenza A, RSV, SARS-CoV-2); Atypical pathogens
Estimated Proportion	**~**35-50% (pathogen-negative)	**~**50-65% (pathogen-positive)
Key Initiating Signals	DAMPs (eHsp70, ATP, HMGB1, S100 proteins, mtDNA, uric acid, LL37-nucleic acid complexes)	PAMPs (LPS, LTA, viral RNA/DNA, flagellin, bacterial CpG DNA)
Primary PRRs Engaged	TLR2/TLR4 (for some DAMPs), RAGE, P2X7 (for ATP), intracellular sensors (e.g., cGAS for mtDNA)	TLR2 (Gram+ bacteria), TLR4 (LPS), TLR3/TLR7/TLR8 (viral RNA), TLR9 (CpG DNA), NOD1/NOD2 (bacterial peptidoglycan)
NLRP3 Activation Mechanism	Signal 1 (priming): NF-κB activation via DAMP-TLR/RAGE signaling; Signal 2 (activation): K^+^ efflux (ATP-P2X7), mitochondrial dysfunction, ROS generation, lysosomal disruption	Signal 1 (priming): NF-κB activation via PAMP-TLR signaling; Signal 2 (activation): Bacterial toxins, viral proteins (e.g., IAV M2), K^+^ efflux, ROS, lysosomal disruption
Key Downstream Effectors	IL-1β, IL-18, Caspase-1, Gasdermin D (pyroptosis)	IL-1β, IL-18, Caspase-1, Gasdermin D (pyroptosis); Additionally: Type I IFNs (antiviral response), IL-6, TNF-α (broader antimicrobial response)
Immune Cell Dynamics	Predominantly: Macrophages (M1 polarization), damaged epithelial cells, neutrophils (NETosis)	Mixed: Macrophages, neutrophils, dendritic cells, T-cells (adaptive immunity engagement); Viral-specific CD8^+^ T cell response
Diagnostic Biomarkers	Elevated serum DAMPs (eHsp70, HMGB1); Negative microbiological tests; CRP and NLR may be moderately elevated	Positive culture/PCR; Elevated procalcitonin (bacterial); Viral antigens/RNA; CRP and NLR often significantly elevated
Corticosteroid Response	Generally more responsive (sterile inflammation driven by DAMPs often corticosteroid-sensitive)	Variable: Effective in viral-induced inflammation; Less effective in bacterial exacerbations (may impair bacterial clearance)
Potential Targeted Therapies	NLRP3 inhibitors (MCC950, CY-09, Dapansutrile); DAMP-neutralizing agents (anti-HMGB1, soluble RAGE); P2X7 antagonists	Antimicrobials; Immunomodulators targeting specific PAMPs; Vaccination strategies (preventive)
Exacerbation Severity	Mild to moderate, depending on DAMP load and host susceptibility	Mild to severe; Higher risk of severe exacerbations requiring ICU admission (especially viral)
Prognostic Implications	Recurrent sterile exacerbations may indicate persistent tissue damage/remodeling	Recurrent infectious exacerbations may indicate impaired host defense or microbiome dysbiosis

This mechanistic divergence has direct clinical consequences. Sterile exacerbations may be more responsive to corticosteroid therapy and represent an ideal target population for emerging NLRP3-specific inhibitors. In contrast, infectious exacerbations require careful antimicrobial stewardship—antibiotics are essential for bacterial episodes but may be unnecessary or even harmful in viral-predominant cases. Herein lies a major clinical challenge: current diagnostic criteria cannot reliably exclude low-burden or unculturable pathogens. For instance, one study identified a strong association between high sputum IL-1β and bacterial presence (OR = 9), yet nearly half of AECOPD cases lacked any detectable pathogen despite comprehensive culture-based screening (27). This implies that either “sterile” inflammation can phenocopy infection, or current microbiological methods fail to capture relevant microbial triggers—underscoring the urgent need for host-response–based classifiers that integrate immune signatures rather than relying solely on pathogen detection. Notably, viral infections, particularly influenza A virus, can amplify sterile inflammation by inducing robust DAMP release, creating a “mixed phenotype” exacerbation where PAMP and DAMP signals synergize to drive hyperinflammation (33). Therefore, clarifying these distinctions is essential for advancing precision medicine in AECOPD.

### 2.5 Molecular mechanisms of DAMPs in aseptic AECOPD

A diverse array of DAMPs has been implicated in the pathogenesis of sterile AECOPD, each engaging specific receptors and triggering distinct downstream signaling cascades. As summarized in **Table 2**, these endogenous molecules originate from various cellular sources—including stressed epithelial cells, activated immune cells, and damaged mitochondria—and exert their effects through pattern recognition receptors such as TLRs, RAGE, and P2X7. Notably, several DAMPs exhibit cell type-specific effects: for example, eHsp70 activates the NLRP3 inflammasome in monocytes while exerting regulatory functions in bronchial epithelial cells. The convergence of multiple DAMP signals on common downstream pathways, particularly the NLRP3 inflammasome, underscores the potential of targeting these molecules or their receptors for therapeutic intervention in AECOPD.

**Table 2:** Major DAMPs involved in sterile AECOPD: receptors, downstream pathways, and cellular targets.

DAMP type	Source/Release mechanism	Primary receptors	Downstream signaling pathways	Key cellular targets	Pathological effects in AECOPD
eHsp70 (extracellular Hsp70)	Released from stressed/damaged epithelial cells under oxidative stress, cigarette smoke exposure, hypoxia	TLR2, TLR4, CD91, CD40	NF-κB activation; MAPK pathway; Cell type-dependent NLRP3 modulation	Monocytes, macrophages, bronchial epithelial cells	Dual immunomodulatory role: pro-inflammatory in monocytes (↑IL-1β), regulatory in epithelial cells; Promotes ATP release
ATP	Released from injured cells via pannexin/connexin channels; Mechanical stress; Cell lysis	P2X7 (ligand-gated ion channel)	K^+^ efflux → NLRP3 inflammasome assembly → Caspase-1 activation → IL-1β/IL-18 maturation; Pyroptosis	Macrophages, airway epithelial cells, neutrophils	Primary driver of NLRP3 activation; Amplifies inflammatory cascade; Promotes pyroptosis and tissue damage
HMGB1	Passive release from necrotic cells; Active secretion by activated immune cells	TLR2, TLR4, RAGE	NF-κB activation; MAPK/ERK; Autophagy regulation	Macrophages, epithelial cells, neutrophils	Promotes cytokine release; Induces autophagy; Amplifies sterile inflammation; Correlates with exacerbation severity
S100 proteins (S100A8/A9, S100A12)	Secreted by phagocytes; Released from damaged epithelial cells	TLR4, RAGE	NF-κB activation; MAPK pathway; NADPH oxidase → ROS generation	Neutrophils, macrophages, endothelial cells	Promote neutrophil recruitment; Amplify oxidative stress; Contribute to NETosis
mtDNA	Released from damaged mitochondria during cellular stress; Oxidative stress-induced mitochondrial dysfunction	TLR9 (endosomal), cGAS (cytosolic)	TLR9-MyD88-NF-κB; cGAS-STING-IRF3/NF-κB; NLRP3 co-activation	Macrophages, dendritic cells, epithelial cells	Triggers type I IFN response; Co-activates NLRP3 inflammasome; Links mitochondrial dysfunction to inflammation
Uric acid	Released from necrotic cells; Generated from purine metabolism	Phagocytic receptors (indirect); Forms MSU crystals	Crystal phagocytosis → lysosomal disruption → cathepsin release → NLRP3 activation	Macrophages, dendritic cells	Forms monosodium urate (MSU) crystals; Activates NLRP3 via lysosomal damage; Promotes Th17 responses
IL-33	Released from damaged epithelial and endothelial cells; Necrosis	ST2 (IL-33R)	MyD88-IRAK-TRAF6-NF-κB; MAPK activation	ILC2s, Th2 cells, mast cells, eosinophils	Alarmin cytokine; Promotes type 2 immunity; Amplifies airway inflammation; Links tissue damage to immune activation
Self-DNA/RNA	Released from necrotic or apoptotic cells; NETosis	TLR7/8 (RNA), TLR9 (DNA), cGAS (cytosolic DNA), RIG-I/MDA5 (cytosolic RNA)	TLR-MyD88-NF-κB; cGAS-STING-TBK1-IRF3; RIG-I-MAVS-IRF3/7	Macrophages, dendritic cells, epithelial cells	Triggers type I IFN response; Co-activates inflammasomes; Amplifies autoimmune-like inflammation

#### 2.5.1 eHsp70: a context-dependent DAMP and potential acute exacerbation trigger

It is crucial to contextualize the role of eHsp70 within the spectrum of DAMP biology in COPD (46). *In vitro* and acute-phase models robustly support its immunomodulatory functions (57). However, its reliability as a systemic biomarker in stable, severe COPD may be limited, as suggested by clinical data showing low detection rates in patient sera (58). This observation implies that the pathophysiological contribution of eHsp70 is likely more relevant to immune modulation within the local airway microenvironment during acute stress, rather than serving as a circulating indicator of systemic chronic inflammation (50, 59). This aligns with the emerging concept that different DAMPs can play distinct roles, with some acting as chronic markers of disease severity and others as acute triggers of exacerbations.

EHsp70 serves as a critical DAMP released into the extracellular milieu in response to various cellular stressors, including oxidative stress, hypoxia, and cigarette smoke exposure—key factors implicated in the pathogenesis of AECOPD. These triggers, such as oxidative stress and infection, are potent inducers of cellular damage and often contribute to the onset of acute exacerbations. The release of eHsp70 under these conditions indicates that it functions not as a constitutive marker of stable disease, but rather as a dynamic damage signal within the “event cascade” of an acute exacerbation (60). Following its release into the extracellular milieu, eHsp70 modulates immune responses through interactions with various immune cells, notably by exerting context-dependent regulatory effects on the NLRP3 inflammasome, a central mediator of IL-1β-driven inflammation (61). This duality enables eHsp70 to act either as an activator or inhibitor of NLRP3, positioning it as a pivotal yet ambivalent immunomodulator whose functional outcome is determined by the microenvironment and the responding immune cell repertoire. The ability of eHsp70 to activate or inhibit the NLRP3 inflammasome is notably cell-type dependent. For instance, *in vitro* studies have shown that in monocytic cells, eHsp70 can enhance the activation of the NLRP3 inflammasome, leading to increased IL-1β secretion and ATP release, which further propagates inflammation. Conversely, experiments using bronchial epithelial cells suggest that eHsp70 may exert antagonistic effects on NLRP3 activation, potentially serving as a regulatory mechanism to prevent excessive inflammation in the airway. This duality in the response to eHsp70 emphasizes the complexity of immune interactions and the importance of cellular context in determining the outcome of inflammation. The differential effects of eHsp70 on ATP release and IL-1β expression also suggest that it plays a critical role in shaping the local immune environment within the airways, which is particularly relevant during AECOPD (62).

The observed dichotomy—wherein eHsp70 triggers NLRP3 inflammasome assembly in myeloid lineages (e.g., monocytes and macrophages) yet suppresses it in bronchial epithelial cells—is not an experimental artifact. Instead, this likely represents a functional specialization: professional immune cells are evolutionarily tuned to amplify danger cues and launch potent inflammatory cascades, whereas structural barrier cells safeguard tissue homeostasis by curbing excessive inflammasome activity. Nevertheless, current evidence relies heavily on immortalized models, such as THP-1 monocytes and NCI-H292 bronchial epithelial cells, which may inadequately mirror the receptor landscapes, signaling dynamics, or metabolic profiles of primary human airway cells *in vivo*. Consequently, although this cell-type-specific mechanism is biologically plausible, its quantitative significance and consistency in patients experiencing AECOPD remain unverified, warranting validation via single-cell RNA sequencing or spatial transcriptomics of airway specimens obtained during exacerbation events.

The “cell-type dependent” duality of eHsp70 may stem from the intrinsic functional programming of different receptors involved and responsive cells. In professional inflammatory cells like monocytes and macrophages, eHsp70 likely functions as a canonical alarmin by engaging pro-inflammatory receptors such as TLR4, delivering a potent NF-κB-mediated priming signal that upregulates NLRP3 and pro-IL-1β, and may synergize with cellular stress to facilitate full inflammasome assembly and pyroptosis. This aligns with the role of other DAMPs like HMGB1 and S100 proteins in amplifying sterile inflammation in myeloid cells. Conversely, in structural barrier cells like bronchial epithelium, eHsp70 may engage a different receptor repertoire that steers the signal toward cytoprotective, repair, or anti-inflammatory pathways. This can result in a de facto inhibition of NLRP3 by failing to provide adequate priming, inducing negative regulators, or promoting competing homeostatic processes. In addition, the interplay between eHsp70 and other DAMPs, such as ATP, further complicates the immune response landscape. The release of ATP in conjunction with eHsp70 can amplify the inflammatory response by activating purinergic receptors on immune cells, thus enhancing the secretion of pro-inflammatory cytokines (63). This synergistic effect may contribute to the exacerbation of airway inflammation observed in COPD patients, where both eHsp70 and ATP are elevated during acute episodes. Furthermore, the interaction of eHsp70 with PRRs on immune cells, such as TLRs, adds another layer of complexity to its immunomodulatory role. For instance, eHsp70 can engage Toll-like receptor 4(TLR4), leading to downstream signaling that promotes inflammation (62). Together, these findings position eHsp70 as a central immunomodulatory DAMP whose regulation of the NLRP3 inflammasome and interactions with PRRs offer potential targets for therapeutic intervention in sterile inflammatory diseases such as COPD.

#### 2.5.2 The S100A8/A9-RAGE axis and other clinically relevant DAMPs in AECOPD

Beyond eHsp70, other DAMPs with well-established links to COPD severity and exacerbations merit consideration, as they may serve distinct roles across the COPD disease spectrum (64). The S100A8/A9 is a prototypical DAMP with significant clinical relevance. It is rapidly released by activated myeloid cells (e.g., neutrophils, macrophages) and stressed epithelial cells following tissue injury (65). Acting as a ligand for both the receptor for RAGE and TLR4, S100A8/A9 activates a potent pro-inflammatory signaling cascade (66, 67). This signaling constitutes a key immunoregulatory mechanism for the NLRP3 inflammasome, promoting its assembly and subsequent activation of caspase-1, which in turn leads to maturation of IL-1β and IL-18 as well as pyroptosis (68). This pathway is strongly implicated in amplifying sterile inflammation and has been associated with various autoimmune and inflammatory pathologies. Importantly, the S100A8/A9-RAGE axis has attracted considerable clinical interest for its potential role in non-infective exacerbations of COPD (69). It has a rapid release profile and strong inflammasome activation ability, positioning it as a plausible acute trigger within the exacerbation cascade. This directly links tissue damage to the fulminant inflammatory response characteristic of an acute event (70).

In contrast, nucleic acid DAMPs, such as mtDNA, represent a distinct category. Released from damaged cells and mitochondria, mtDNA serves as a potent DAMP, activating the NLRP3 inflammasome as well as the cGAS-STING pathway (71). Its presence tends to be more chronically sustained, reflecting cumulative cellular damage and mitochondrial dysfunction (72). Thus, while mtDNA and similar molecules contribute to the inflammatory milieu, they primarily function as chronic markers of disease severity and progression risk, rather than as specific triggers of an acute exacerbation. This functional dichotomy—between acute exacerbation triggers (e.g., S100A8/A9) and chronic severity markers (e.g., mtDNA)—highlights the heterogeneity of DAMP involvement in AECOPD pathophysiology. The functional dichotomy positioning S100A8/A9 as an acute instigator and mtDNA as a marker of chronic severity constitutes one of the most robust conceptual paradigms within contemporary DAMP biology. This framework harmonizes ostensibly contradictory findings in the literature: investigations targeting exacerbation forecasting consistently highlight S100A8/A9 (69), while those assessing long-term mortality or disease trajectory prioritize mtDNA (72). Nevertheless, a crucial limitation emerges regarding sampling timing; mtDNA levels can also spike acutely after substantial tissue damage, such as severe trauma or burns (50). This indicates that categorizing mtDNA as a “chronic” DAMP is primarily valid within the setting of stable COPD. During active AECOPD episodes, transient mtDNA elevations may arise secondary to acute cellular necrosis, potentially obscuring interpretation if samples are collected without precise clinical staging. Consequently, future research must stratify DAMP assessments by exacerbation phase to prevent the misassignment of biological functions.

HMGB1 is another archetypal DAMP with a well-documented role in sterile inflammation and fibrosis across various organ systems (73). HMGB1 is normally a nuclear protein that translocates to the cytoplasm and is released extracellularly upon cellular stress or damage. It functions as a potent alarmin by engaging multiple receptors, including TLR4 and the receptor for RAGE (74). The HMGB1/TLR4 axis has been shown to promote the activation of pancreatic stellate cells and extracellular matrix deposition, contributing to fibrosis in chronic pancreatitis (75). This process is indicative of its role in driving chronic, fibrotic tissue remodeling. This pathway promotes autophagy and NLRP3 inflammasome activation, creating a feed-forward loop of inflammation and fibrosis (76). In the context of COPD, HMGB1 released from damaged airway cells may similarly engage these pathways, potentially contributing to both the acute inflammatory burst during exacerbations and the chronic, progressive airway remodeling that characterizes the disease.

#### 2.5.3 ATP as a signaling molecule mediating inflammasome activation

ATP functions as a key DAMP that facilitates inflammasome activation primarily through P2X7 receptor signaling. Released into the extracellular milieu upon cellular injury, ATP binds to the P2X7 receptor—a ligand-gated ion channel—triggering potassium (K+) efflux. This ionic shift constitutes an essential upstream signal for NLRP3 inflammasome assembly, ultimately promoting caspase-1 activation and the maturation of IL-1β and IL-18 (77). *In vitro* experiments suggest that the magnitude of this inflammatory response is modulated by both the concentration and temporal pattern of ATP release, with sustained or elevated ATP levels driving robust inflammasome activity. Importantly, ATP-P2X7-mediated signaling establishes a critical communication axis between injured airway epithelial cells and immune cells, shaping the inflammatory microenvironment during sterile exacerbations in COPD (78).

Furthermore, the role of ATP in inflammasome activation extends beyond mere initiation; it is intricately linked to the regulation of inflammation intensity. The concentration of ATP released during cellular stress can dictate the strength of the inflammatory response. High levels of ATP can lead to excessive inflammation, contributing to tissue damage and exacerbating conditions such as COPD, while lower levels may promote a more controlled inflammatory response (79). This regulatory mechanism is particularly relevant in the context of airway epithelial cells, which, upon sensing damage, release ATP to communicate with resident immune cells, thereby orchestrating a localized inflammatory response. The interplay between ATP levels and the timing of its release is crucial for maintaining homeostasis within the airway environment, as dysregulated ATP signaling can lead to chronic inflammation and tissue remodeling (80).

In the context of sterile inflammation, ATP-mediated signaling is recognized as a significant driver of the inflammatory response during AECOPD. The activation of the NLRP3 inflammasome by ATP not only leads to the production of pro-inflammatory cytokines but also promotes pyroptosis, a form of programmed cell death that further amplifies inflammation (81). This process is particularly relevant in COPD, where the balance between protective and pathological inflammation is critical for disease progression. The ability of ATP to modulate the inflammasome response highlights its potential as a therapeutic target; strategies aimed at regulating ATP levels or blocking its interaction with the P2X7 receptor could offer new avenues for managing inflammation in COPD and other related conditions (82).

#### 2.5.4 Cell type-specific differences in response to DAMPs

The response to DAMPs is notably distinct across various cell types, particularly when comparing monocyte-derived macrophages and airway epithelial cells. Macrophages, which are key players in the immune response, exhibit a robust activation of the NLRP3 inflammasome upon exposure to DAMPs. This activation results in the secretion of pro-inflammatory cytokines, such as IL-1β and IL-18, which amplify the inflammatory response and contribute to tissue damage in conditions like COPD (83). In contrast, airway epithelial cells display a more nuanced response to DAMPs, particularly HMGB1 and eHsp70. These epithelial cells can exhibit an inhibitory effect on inflammation, potentially through the modulation of signaling pathways that suppress the NLRP3 inflammasome activation. This dichotomy in response underscores the complexity of immune regulation in the lung, where epithelial cells may act to limit excessive inflammation while macrophages promote it, thereby influencing the overall inflammatory milieu and the extent of tissue injury (55).

The differential responses of these cell types to DAMPs also highlight the significance of the microenvironment in shaping immune outcomes. For instance, the presence of specific cytokines and the overall cellular context can dictate whether macrophages adopt a pro-inflammatory M1 phenotype or a more reparative M2 phenotype in response to DAMPs (84). In the context of COPD exacerbations, the interplay between macrophages and epithelial cells becomes particularly critical. Epithelial cells, when damaged, release DAMPs that can recruit and activate macrophages, leading to a feedback loop of inflammation that exacerbates tissue injury. Conversely, if epithelial cells are able to effectively modulate the response to DAMPs, they may mitigate the inflammatory response and promote healing (85).

Furthermore, the complexity of intercellular interactions cannot be overstated. The communication between macrophages and epithelial cells is mediated through various signaling molecules, including cytokines and chemokines, which can alter the functional state of both cell types. For example, macrophages can produce transforming growth factor-beta (TGF-β), which has been shown to influence epithelial cell behavior, promoting repair and regeneration processes (86). Conversely, epithelial cells can release factors that modulate macrophage polarization, potentially steering them towards a more anti-inflammatory phenotype. This intricate network of signaling and feedback mechanisms plays a crucial role in determining the overall inflammatory response and tissue repair processes in the lungs during AECOPD (87).

#### 2.5.5 The synergistic activation mechanism of DAMPs and PAMPs

The interplay between PAMPs and DAMPs is crucial in the activation of the NLRP3 inflammasome, particularly in the context of AECOPD. *In vitro* studies using immune cells have shown that bacterial components such as LPS and lipoteichoic acid (LTA) synergize with DAMPs like eHsp70 and ATP to enhance NLRP3 inflammasome activation (88). This co-activation leads to a robust inflammatory response characterized by increased secretion of IL-1β and ATP, exacerbating the inflammatory milieu during AECOPD. The mechanisms underlying this synergistic effect involve the simultaneous recognition of both PAMPs and DAMPs by PRRs, which trigger distinct yet overlapping signaling pathways that culminate in the activation of the NLRP3 inflammasome. For instance, LPS and LTA, through their interaction with TLRs, initiate pro-inflammatory signaling cascades that sensitize immune cells to subsequent DAMP exposure, thereby amplifying the inflammatory response (89).

The synergistic effects of DAMPs and PAMPs extend beyond mere activation of the NLRP3 inflammasome. They also lead to a significant increase in the release of pro-inflammatory cytokines, particularly IL-1β, which plays a pivotal role in promoting inflammation and tissue damage during AECOPD (90). The release of ATP, another DAMP, further contributes to this inflammatory cascade by acting as a signaling molecule that can activate purinergic receptors on immune cells, thereby enhancing their activation and recruitment to the site of inflammation. This dual action of DAMPs and PAMPs create a feedback loop that not only sustains but also amplifies the inflammatory response, leading to a vicious cycle of inflammation and tissue injury that characterizes AECOPD patients (91).

Notably, the interaction between DAMPs and PAMPs reveals a complex network of inflammation that intertwines infectious and sterile factors. In the context of COPD, this interplay suggests that exacerbations may not solely be driven by infectious agents but also by endogenous signals from damaged tissues that promote inflammation. This understanding challenges the traditional view of infection-driven exacerbations and highlights the importance of targeting both PAMPs and DAMPs in therapeutic strategies aimed at mitigating inflammation and improving outcomes in COPD patients.

However, the majority of evidence supporting DAMP-PAMP synergy is derived from *in vitro* co-stimulation experiments that often employ supraphysiological concentrations of inducers. In clinical settings of AECOPD, microbial loads are typically low, and DAMP concentrations fluctuate dynamically over time. It remains unclear whether synergistic inflammasome activation occurs at clinically relevant concentrations or whether a single signal predominates under physiological conditions. Moreover, viral PAMPs—for example, double-stranded RNA—engage different pattern recognition receptors such as RIG-I or MDA5, and may produce distinct synergy patterns compared to bacterial LPS; however, this nuance is rarely addressed in current literature. Thus, while the principle of synergy is well established in reductionist models, its quantitative contribution to the heterogeneity of human AECOPD requires further investigation in well-phenotyped patient cohorts.

#### 2.5.6 The impact of inflammasome activation on airway structure and function in COPD

The activation of the NLRP3 inflammasome plays a pivotal role in mediating the inflammatory response in COPD, particularly through the release of pro-inflammatory cytokines that exacerbate airway inflammation and facilitate tissue remodeling. NLRP3 inflammasome activation is triggered by various stimuli, including DAMPs released during cellular stress and injury, which are prevalent in the COPD context due to chronic exposure to cigarette smoke and other irritants (92). The resultant cytokine release, particularly IL-1β and IL-18, leads to a cascade of inflammatory responses that promote the recruitment of immune cells to the airways, thereby intensifying inflammation. This inflammatory milieu not only contributes to the pathogenesis of COPD but also drives the processes of airway remodeling and structural changes, including epithelial cell hyperplasia, mucus hypersecretion, and fibrosis, which collectively impair lung function (93).

Chronic exposure studies in animal models of COPD have demonstrated that long-term activation of the NLRP3 inflammasome has significant implications for airway structure and function, leading to progressive airway fibrosis, increased mucus secretion, and exacerbated airway obstruction (94). The persistent inflammatory environment fosters a cycle of injury and repair that ultimately results in maladaptive remodeling of the airway tissues. In COPD, the excessive deposition of extracellular matrix components, such as collagen, contributes to airway wall thickening and narrowing, which is a hallmark of the disease. This remodeling process is further exacerbated by the chronic presence of inflammatory cells, including neutrophils and macrophages, which release proteolytic enzymes and ROS that damage the airway epithelium and promote fibrosis (95). As a result, patients experience increased airway resistance, reduced airflow, and impaired gas exchange, leading to the characteristic symptoms of COPD, such as dyspnea and chronic cough (96).

Moreover, the inflammasome serves as a critical nexus linking immune activation to tissue damage in COPD. The NLRP3 inflammasome not only facilitates the release of pro-inflammatory cytokines but also contributes to a form of programmed cell death known as pyroptosis. This process is characterized by the formation of pores in the cell membrane, leading to cell lysis and the subsequent release of intracellular contents that further amplify the inflammatory response. Pyroptosis of airway epithelial cells and alveolar macrophages has been shown to exacerbate lung inflammation and contribute to the progression of COPD based on these preclinical findings (97). The interplay between inflammasome activation, cytokine release, and pyroptosis underscores the importance of targeting these pathways for therapeutic interventions aimed at mitigating airway inflammation and remodeling in COPD (98). Understanding the mechanisms by which inflammasome activation impacts airway structure and function is crucial for developing targeted therapies that can effectively address the underlying pathophysiology of COPD and improve patient outcomes.

Although murine preclinical investigations robustly substantiate the causal involvement of NLRP3 inflammasome activation in promoting airway remodeling (94), direct validation within human subjects remains predominantly indirect. The majority of clinical inquiries have identified correlations between IL-1β concentrations in sputum or serum and radiological or functional indices of disease severity, including computed tomography-derived airway wall thickness or the rate of FEV1 deterioration. Nevertheless, such associations fail to confirm causality, as they are susceptible to confounding variables such as smoking history, advancing age, comorbid conditions, or concurrent infections (27, 29). Moreover, definitive molecular indicators of pyroptosis, notably cleaved gasdermin D, have not undergone systematic quantification in lung specimens obtained from COPD patients spanning diverse disease stages. Consequently, while the inflammasome-remodeling axis constitutes a persuasive therapeutic premise, its pivotal role in human COPD pathogenesis—distinct from being merely one element among numerous parallel inflammatory cascades—awaits conclusive demonstration.

### 2.6 Virus infection-induced NLRP3 inflammasome activation and AECOPD

#### 2.6.1 The role of influenza A virus in AECOPD

Influenza A virus (IAV) infection is a prominent trigger of AECOPD, substantially elevating associated morbidity and mortality. The underlying pathophysiology involves NLRP3 inflammasome activation, which drives a pronounced inflammatory cascade. Following IAV infection, NLRP3 activation leads to marked upregulation of pro-inflammatory cytokines such as IL-1β and IL-18, generating a cytokine storm that intensifies airway inflammation and further compromises already impaired lung function in COPD patients (99). Moreover, airway epithelial cells in COPD exhibit heightened susceptibility to IAV, resulting in more severe and sustained inflammatory responses compared to those in individuals without COPD. This increased vulnerability is linked to pre-existing airway inflammation and structural remodeling, which collectively impair epithelial antiviral defense mechanisms (100).

The persistent inflammatory milieu in COPD establishes a permissive environment for respiratory viral infections, thereby exacerbating airway injury and accelerating functional decline. Following IAV infection, not only is the pre-existing inflammatory cascade amplified, but virus-induced cellular stress and epithelial apoptosis further contribute to progressive lung function deterioration. Clinical studies confirm that IAV infection in COPD patients is associated with significant reductions in both forced expiratory volume in one second (FEV1) and forced vital capacity (FVC), reflecting the profound negative impact of viral pathogens on respiratory mechanics (101). Furthermore, IAV infection elevates susceptibility to secondary bacterial pneumonia, a complication that substantially increases exacerbation severity and hospital admission rates (102).

The clinical impact of IAV in COPD exacerbations is often compounded by co-infection with other respiratory pathogens; for instance, concurrent infection with non-typeable Haemophilus influenzae synergistically amplifies pulmonary inflammation and injury, resulting in more severe disease manifestations (103). Mechanistically, IAV drives acute worsening through NLRP3 inflammasome activation and the ensuing cytokine release, which intensifies the pre-existing inflammatory milieu and airway structural compromise in COPD. This dysregulated immune environment not only accelerates lung function decline but also heightens vulnerability to secondary infections. Consequently, implementing effective preventive strategies—including annual influenza vaccination—remains essential for mitigating exacerbation frequency and improving long-term outcomes in the COPD population (104).

While the association between IAV and adverse outcomes in COPD is well documented, the causal attribution of these effects specifically to NLRP3 inflammasome activation in humans remains inferential. Much of the mechanistic evidence derives from animal models or *in vitro* systems that may not fully recapitulate the immunosenescent, remodeled, and microbiota-altered airway environment of elderly COPD patients. For example, the rat models cited to support NLRP3’s centrality (105) typically use young, otherwise healthy animals exposed to IAV in isolation, whereas human AECOPD often occurs in the context of chronic colonization by bacteria such as H. influenzae or M. catarrhalis (56). This discrepancy raises an important question: does IAV act primarily as a direct inflammasome activator, or does it instead disrupt epithelial barrier integrity, thereby enabling bacterial products (such as LPS or lipoteichoic acid) to access intracellular sensors and drive NLRP3 activation secondarily? The observed synergy between IAV and H. influenzae (103) supports the latter model, suggesting that in real-world AECOPD, viral and bacterial triggers may be inextricably linked, and that exclusively viral NLRP3 activation might be less common than hybrid PAMP-DAMP signaling.

#### 2.6.2 Mechanisms of NLRP3 inflammasome activation induced by IAV

IAV infection serves as a potent inducer of NLRP3 inflammasome activation, a key innate immune mechanism responsible for processing and releasing pro-inflammatory cytokines, including IL-1β and IL-18. This activation follows a coordinated sequence initiated by viral recognition and intracellular stress signaling. During IAV infection, inflammasome assembly proceeds through the recruitment of ASC and pro-caspase-1, leading to proteolytic cleavage of pro-IL-1β and pro-IL-18 into their biologically active forms (106). The process is further regulated by signaling cascades such as RAF-, MEK1/2-, ERK1/2, and AP-1, which augment pro-IL-1β transcription in response to viral challenge (107). Moreover, IAV induces dispersion of the trans-Golgi network (TGN), a structural reorganization that promotes NLRP3 recruitment and facilitates inflammasome complex formation.

The intracellular replication of IAV generates critical stress signals, including mitochondrial dysfunction and ROS production, which are integral to NLRP3 inflammasome activation. Mitochondria-derived ROS, in particular, facilitate the conformational changes required for NLRP3 oligomerization (108). Moreover, viral components such as the IAV M2 protein contribute to NLRP3 activation by modulating host ionic homeostasis. The ion channel function of M2 is essential for promoting potassium efflux—a key upstream signal that triggers inflammasome assembly.

In the context of therapeutic intervention, the selective NLRP3 inhibitor MCC950 has demonstrated efficacy in attenuating IAV−driven hyperinflammation. Experimental studies indicate that MCC950 administration substantially suppresses the expression of NLRP3 inflammasome components while reducing IL−1β and IL−18 release, resulting in mitigated lung injury and improved survival in IAV infection models (105). These findings underscore the therapeutic relevance of targeting the NLRP3 inflammasome to regulate excessive inflammatory responses associated with viral exacerbations, particularly in conditions such as AECOPD.

#### 2.6.3 Validation studies of NLRP3 inflammasome in COPD animal models

Preclinical studies have established the NLRP3 inflammasome as a central mediator in COPD pathogenesis, with IAV challenge in rat models significantly upregulating NLRP3 expression and driving the secretion of key inflammatory cytokines such as IL-1β and IL-18 (105). This NLRP3-driven response amplifies baseline airway inflammation and accelerates functional deterioration, thereby exacerbating disease severity. Therapeutically, selective NLRP3 inhibition has demonstrated substantial efficacy; MCC950 administration effectively suppresses inflammasome activation, reduces inflammatory cytokine levels, and improves lung function and survival outcomes in COPD animal models (109). These findings not only validate NLRP3 as a mechanistically relevant therapeutic target but also support the translational potential of inflammasome-targeted interventions for managing acute exacerbations in clinical COPD.

Animal studies have further delineated the mechanistic pathways linking NLRP3 inflammasome activation to COPD pathology, revealing its role in driving pyroptosis—an inflammatory form of programmed cell death that amplifies tissue injury (110). Activation of NLRP3 by environmental and infectious stimuli, including cigarette smoke and viral pathogens, sustains a cycle of cytokine release and progressive lung damage that worsens COPD symptomatology. The upregulation of NLRP3 following IAV infection, together with the observed efficacy of pharmacological inhibitors such as MCC950 in preclinical models, underscores the inflammasome’s potential as a viable therapeutic target for modulating disease progression in COPD.

Although MCC950 demonstrates robust efficacy in rodent systems, extrapolating these findings to human AECOPD warrants considerable caution. Firstly, prevailing murine COPD models rely exclusively on cigarette smoke exposure, thereby failing to recapitulate the intricate comorbidity landscape—such as cardiovascular pathology and metabolic syndrome—and the polypharmacy regimens characteristic of the elderly clinical population (21). Secondly, experimental protocols typically administer MCC950 either prophylactically or immediately post-infection; such timing is clinically impractical, given that therapeutic intervention in practice commences only after symptom manifestation. Thirdly, and most critically, there is currently no evidence confirming uniform NLRP3 inflammasome upregulation across all AECOPD endotypes. Emerging clinical datasets indicate that this pathway drives only a specific subset of exacerbations, notably those characterized by elevated sputum IL-1β levels concurrent with bacterial detection. Consequently, indiscriminate NLRP3 blockade may confer benefits to merely a fraction of patients while subjecting others to unwarranted immunosuppressive risks. This underscores a pivotal translational gap: the absence of validated biomarkers capable of stratifying AECOPD cohorts based on inflammasome activity prior to the initiation of targeted therapies.

#### 2.6.4 Changes in NLRP3 inflammasome-related biomarkers in clinical patients

Clinical studies have demonstrated that elevated serum and bronchoalveolar lavage fluid levels of IL-1β and IL-18 during AECOPD serve as key biomarkers of NLRP3 inflammasome activation, a central driver of inflammatory responses in exacerbations. These cytokine elevations correlate with disease severity and systemic inflammation, indicating their utility in monitoring disease activity and progression (111). Notably, this increase reflects an underlying pathological process wherein NLRP3 activation by common triggers—such as environmental pollutants and respiratory infections—contributes directly to exacerbation pathogenesis rather than merely accompanying clinical deterioration (112).

The activity of the NLRP3 inflammasome correlates directly with AECOPD severity, where elevated levels of IL-1β and IL-18 are associated with more severe clinical presentations and poorer prognoses. Monitoring these biomarkers provides valuable insights into the inflammatory state of patients, supporting their utility in guiding targeted anti-inflammatory interventions and in stratifying exacerbation risk for individualized management (113). Furthermore, serial assessment of IL-1β and IL-18 can serve as a dynamic tool for evaluating therapeutic efficacy; a decline following treatment suggests a favorable response, whereas persistent elevation may indicate the need for therapeutic adjustment (114). Collectively, NLRP3-associated biomarkers, especially IL-1β and IL-18, represent clinically relevant indicators of disease severity and treatment response in AECOPD.

However, the interpretation of IL-1β and IL-18 as specific readouts of NLRP3 activity is not without limitations. Both cytokines can be processed by proteases other than caspase-1—for instance, neutrophil elastase or proteinase-3—which are abundant in the COPD airway during bacterial exacerbations (29). Therefore, elevated IL-1β in sputum does not unequivocally implicate the NLRP3 inflammasome; it could equally reflect neutrophil-dominated, caspase-1-independent inflammation. This ambiguity is reinforced by a longitudinal sputum study showing that IL-1β elevation during AECOPD strongly associates with bacterial presence but shows no correlation with classical eosinophilic or Th2 markers. Such findings suggest that “IL-1β-high” AECOPD may define a distinct bacterial-inflammatory endotype, rather than a universal viral-NLRP3 phenotype. Consequently, using IL-1β alone as a surrogate for NLRP3 activation risks misclassifying patients and could confound clinical trial outcomes if not paired with more specific markers—such as caspase-1 activity, ASC speck formation, or gasdermin D cleavage—that directly report inflammasome assembly.

#### 2.6.5 The interaction between viral infections and sterile inflammation

Viral infections stimulate the release of DAMPs, which are endogenous molecules signaling cellular injury and amplifying sterile inflammatory responses. In the context of viral pathogens, DAMP release—such as extracellular DNA and RNA—potentiates immune activation by engaging innate sensing mechanisms, notably the NLRP3 inflammasome, leading to enhanced production of pro−inflammatory cytokines including IL−1β and IL−18 (115). This amplification cascade can precipitate a cytokine storm, a dysregulated hyperinflammatory state associated with extensive tissue injury and multi−organ dysfunction.

Overactivation of the immune system in response to viral infection can lead to dysregulated inflammation and substantial tissue damage, a phenomenon particularly prominent in severe cases manifesting as a “cytokine storm.” This hyperinflammatory state involves collateral host tissue injury during antiviral clearance, frequently contributing to complications such as acute respiratory distress syndrome (ARDS) and multi-organ failure (116). The interaction between viral components, DAMPs, and immune signaling highlights the complexity of infection-associated inflammation. For instance, in COVID-19, virus-induced DAMP release exacerbates inflammatory signaling via pathways including NLRP3 inflammasome activation, establishing a self-perpetuating cycle of inflammation and tissue injury. These findings emphasize the critical need for finely tuned immunomodulatory strategies that mitigate excessive inflammation while maintaining effective pathogen clearance (117).

Deciphering the pathways underlying DAMP release and inflammasome activation enables the identification of potential therapeutic targets for immune modulation. Selective intervention in DAMP-sensing or inflammasome signaling pathways could attenuate excessive inflammation while preserving essential antiviral defenses (106, 118). Concurrently, strategies aimed at restoring immune homeostasis—such as the use of targeted anti-inflammatory agents or immunomodulators—may prevent the shift from protective immunity to pathological hyperinflammation, thereby reducing the risk of severe complications associated with dysregulated inflammatory responses.

### 2.7 Latest advances in immune regulation and therapeutic strategies

#### 2.7.1 Preclinical therapeutic strategies: NLRP3 inflammasome inhibitors

The NLRP3 inflammasome represents a central mediator in the pathogenesis of inflammatory diseases, including COPD. Among available inhibitors, MCC950 is distinguished by its selective targeting of NLRP3, demonstrating efficacy in both cellular and animal models. It specifically reduces NLRP3 inflammasome activation, leading to diminished release of key pro-inflammatory cytokines such as IL-1β and IL-18. In preclinical models of COPD exacerbation, MCC950 administration significantly lowers cytokine levels and attenuates acute inflammatory responses (119). Its selectivity against NLRP3—without affecting NLR family CARD domain-containing protein 4 (NLRC4) or absent in melanoma 2 (AIM2) inflammasomes—further supports its potential as a targeted therapeutic (120). Unlike broad-spectrum anti-inflammatory agents, MCC950 inhibits NLRP3 assembly without interfering with upstream signals such as potassium efflux or reactive oxygen species generation, underscoring its unique mechanistic profile and clinical promise in modulating sterile inflammation during COPD exacerbations (121).

Furthermore, preclinical studies have provided compelling evidence that MCC950 can alleviate inflammation related to COPD exacerbations. In various animal models, treatment with MCC950 has been shown to significantly improve lung function and reduce histopathological damage, indicating its potential to translate into clinical benefits for patients suffering from COPD (122). The reduction in inflammatory markers and the protection against lung tissue damage observed in these studies suggest that NLRP3 inhibition could be a viable strategy for managing AECOPD, which are often triggered by environmental factors such as cigarette smoke and viral infections (123, 124). However, while these findings are promising, the clinical application of MCC950 and similar inhibitors necessitates a thorough evaluation of their long-term safety and efficacy in human subjects.

Looking ahead, the future of NLRP3 inflammasome inhibitors like MCC950 in clinical settings hinges on comprehensive studies that assess not only their therapeutic potential but also their safety profiles over extended periods. Current research is focused on understanding the pharmacokinetics and pharmacodynamics of MCC950, as well as its potential side effects, which are critical for establishing its clinical utility (125). Additionally, the exploration of combination therapies that include MCC950 alongside existing treatments for COPD may enhance its efficacy and provide a multifaceted approach to managing this complex disease (112). As insights into the NLRP3 inflammasome’s contribution to inflammatory pathologies advance, the design of increasingly selective and potent inhibitors will be crucial for refining therapeutic approaches aimed at attenuating inflammation and enhancing clinical outcomes in conditions such as COPD.

Despite demonstrating promising efficacy in preclinical studies, the clinical translation of NLRP3 inhibitors for COPD/AECOPD faces unique challenges. Specifically, COPD patients often present with immunosenescence and a persistent ‘inflammatory load’, which may maintain NLRP3 in an active state that differs from that seen in acute infection models and may be more resistant to complete inhibition (126). Second, the existence of MCC950-insensitive NLRP3 mutants highlights the need for developing broader-spectrum or activation-mode-specific inhibitors (18). Third, the safety concern that long-term inhibition of NLRP3—a key innate immune sensor—might exacerbate the already elevated infection risk in COPD patients requires careful consideration (83).

The initial optimism surrounding MCC950 necessitates a rigorous evaluation of the translational disconnect between preclinical models and clinical reality. Currently, the vast majority of supportive evidence originates from studies utilizing young, genetically uniform murine subjects subjected to acute cigarette smoke exposure or isolated pathogen challenges (122). Conversely, the prototypical patient experiencing AECOPD is typically elderly, characterized by immunosenescence, burdened by multiple comorbidities, and colonized by a dysbiotic airway microbiome (25). This profound discrepancy casts significant doubt on whether the NLRP3 inflammasome in human COPD possesses the same pharmacological accessibility observed in rodent systems. Furthermore, the premise that NLRP3 hyperactivity is a ubiquitous feature across all COPD phenotypes is increasingly contested by clinical biomarker investigations. These studies indicate that inflammasome-driven pathology is restricted to a specific subset of exacerbations, notably those complicated by bacterial co-infection and elevated sputum IL-1β levels. Should NLRP3 activation prove not to be a universal hallmark of AECOPD, broad-spectrum inhibition via agents like MCC950 risks failure in unselected clinical trials. Such outcomes would likely stem not from inadequate target engagement, but rather from the absence of target activity in a substantial proportion of the patient population. Consequently, these findings highlight the critical imperative for developing companion diagnostics capable of identifying the “NLRP3-high” endotype prior to therapeutic intervention.

#### 2.7.2 Targeting DAMPs: immune regulatory strategies

The modulation of DAMPs represents a promising avenue for therapeutic intervention in conditions characterized by sterile inflammation, such as AECOPD. DAMPs, including eHsp70 and ATP, play critical roles in the initiation and perpetuation of inflammatory responses. Targeting the release of these molecules, or their signaling pathways, could potentially attenuate inflammation and improve clinical outcomes. For instance, strategies aimed at regulating eHsp70 and ATP release, or their respective receptor signaling pathways, have shown promise in reducing inflammation (127). By inhibiting the receptors that mediate DAMP signaling, such as TLRs and purinergic receptors, it may be possible to dampen the excessive inflammatory responses observed in COPD exacerbations.

Current preclinical research is actively exploring immune-regulatory agents and small molecules designed to selectively inhibit DAMP-activated pathways, thereby mitigating their pro-inflammatory effects. For instance, compounds that specifically suppress NLRP3 inflammasome activation—induced by DAMPs—are under investigation for their potential to alleviate the inflammatory burden during COPD exacerbations. The development of biologics capable of neutralizing specific DAMPs or their receptors offers another promising direction, potentially enabling more precise and tolerable anti-inflammatory strategies (128). Concurrently, personalized medicine approaches that incorporate individual DAMP profiles could guide tailored therapeutic selections, optimizing treatment efficacy by targeting the patient-specific inflammatory drivers (46).

#### 2.7.3 The potential of multi-target combination therapy

The management of COPD, particularly during acute exacerbations, requires a comprehensive therapeutic strategy that addresses the multifactorial nature of the disease. Multi-target combination therapy, integrating antiviral, anti-inflammatory, and immunomodulatory agents, has emerged as a promising approach to simultaneously target diverse pathogenic mechanisms. For example, therapies directed against DAMPs—which drive sterile inflammation—and agents inhibiting specific pathways such as NLRP3 inflammasome activation may complement conventional antiviral treatments (129). This synergistic regimen aims to enhance clinical responses, reduce exacerbation frequency and severity, and improve overall outcomes by concurrently modulating inflammatory and infectious components of the disease (130).

Moreover, the integration of immunomodulatory therapies, such as biologics targeting type 2 inflammation, into combination regimens has shown promise in improving patient outcomes. For example, dupilumab and benralizumab have demonstrated efficacy in reducing exacerbation rates and improving lung function in patients with elevated eosinophil levels, a common feature in certain COPD phenotypes (131). When combined with standard bronchodilators and corticosteroids, these targeted agents exemplify a tailored, multi-target strategy that addresses heterogeneous disease mechanisms. This approach enables more effective management of acute exacerbations, potentially improving patient quality of life and reducing healthcare utilization. The continued development and clinical validation of multi-target combination therapies are crucial for refining COPD management and optimizing outcomes in acute exacerbations (132).

While the scientific justification for combination therapy is robust, it inevitably introduces substantial intricacies into both trial architecture and clinical execution. A fundamental dichotomy persists between the “precision medicine” paradigm, which targets distinct endotypes (e.g., utilizing benralizumab for eosinophilic phenotypes), and the “broad-spectrum” strategy that incorporates NLRP3 or DAMP inhibitors to address non-type 2 inflammatory pathways. Although these approaches are not mutually exclusive, their effective integration demands rigorous patient stratification. Ultimately, the efficacy of multi-target regimens relies heavily on the prior establishment of rapid, point-of-care biomarkers—such as circulating eosinophils, sputum IL-1β, or CRP—to direct rational therapeutic pairing. In the absence of such diagnostic tools, combination therapy risks devolving into a costly, empiric “shotgun” method rather than functioning as a precisely guided intervention.

#### 2.7.4 Biomarker-guided precision medicine

Dynamic monitoring of biomarkers plays a crucial role in the early identification of acute exacerbations and the timely adjustment of treatment plans. For instance, fluctuations in blood eosinophil counts and other inflammatory markers can signal impending exacerbations, prompting proactive management strategies. Research indicates that patients exhibiting high eosinophil counts are more likely to benefit from corticosteroid therapy, thereby reinforcing the importance of biomarker monitoring in clinical practice (133). By employing a treatable traits framework, clinicians can utilize biomarker data to identify modifiable risk factors and tailor interventions accordingly (134). This proactive approach not only mitigates the impact of exacerbations on lung function and quality of life but also reduces healthcare costs associated with hospitalizations and emergency visits.

The development of novel biomarkers is advancing personalized management strategies in COPD by enabling the identification of disease-specific molecular signatures and phenotypes. Multi-omics approaches—including genomics, proteomics, and metabolomics—facilitate the discovery of circulating markers, such as microRNAs and proteins, that not only reflect disease activity but also predict treatment response and progression (135). Further complexity is illustrated by emerging evidence linking gut microbiota and their metabolites to exacerbation risk, underscoring the need for integrative biomarker panels (136). Among key pathophysiological indicators, NLRP3 inflammasome activity and its downstream effectors provide dynamic measures of disease activity, supporting early exacerbation detection and guiding therapeutic adjustments. The continued refinement of biomarker-driven frameworks promises to transform COPD care into a more precise, individualized practice, ultimately improving long-term disease management and patient outcomes.

Although multi-omics approaches and emerging biomarkers hold considerable potential, a realistic evaluation of present constraints is essential. Currently, blood eosinophil count stands as the sole biomarker possessing adequate validation, widespread availability, and cost-efficiency for routine application in COPD management (133). Conversely, candidate indicators reflecting NLRP3 inflammasome activity—specifically caspase-1-cleaved IL-18 or gasdermin D fragments—lack commercially standardized assays and necessitate sophisticated analytical platforms, including mass spectrometry or digital ELISA (39). Likewise, while gut microbiota profiles attract substantial scientific attention, their immediate impact on clinical decision-making remains limited owing to prolonged turnaround times and the absence of established interventional protocols. Consequently, a significant gap persists between biomarkers identified during discovery phases and those ready for clinical implementation. Until reliable point-of-care tests measuring inflammasome activation become available, NLRP3-directed therapeutic strategies will remain predominantly theoretical. In the meantime, practical composite indices integrating C-reactive protein levels, eosinophil counts, and patient clinical history may provide a more attainable pathway toward personalized management of AECOPD.

### 2.8 Translational and clinical perspective: strategies and challenges in targeting the DAMP-NLRP3 axis

Although the pathogenic role of the DAMP-NLRP3 axis in AECOPD is increasingly recognized, translating this knowledge into effective clinical therapies faces systemic hurdles. Current therapeutic strategies primarily focus on intercepting upstream DAMP signaling or inhibiting downstream NLRP3 inflammasome activation directly (137).To better illustrate the translational landscape, this section distinguishes between preclinical-stage strategies (largely supported by *in vitro* and animal model studies) and clinical-stage strategies (those that have entered human trials or are under clinical investigation).

#### 2.8.1 Preclinical-stage strategies

Preclinical studies have developed various interventions targeting the DAMP-NLRP3 axis, most of which remain at the experimental validation stage (138, 139). Upstream strategies aim to neutralize initial damage signals or block their corresponding receptors, such as anti-HMGB1 antibodies, soluble RAGE, and TLR4 or P2X7 antagonists (140). Although these strategies offer potential target specificity, their efficacy is challenged by the redundancy of the DAMP network, where multiple DAMPs can activate common downstream pathways through different receptors (141). Downstream strategies focus on directly inhibiting NLRP3 assembly or activity. The small-molecule inhibitor MCC950, which selectively blocks NLRP3 without affecting NLRC4 or AIM2 inflammasomes, has shown significant efficacy in preclinical models of various inflammatory diseases (125). In animal models of acute exacerbation of COPD, MCC950 administration reduced IL-1β and IL-18 levels and attenuated acute inflammatory responses (105). In addition to MCC950, other investigational compounds have shown NLRP3 inhibitory activity in preclinical studies, and efforts continue to develop more selective and potent inhibitors (142). Emerging preclinical strategies also target gasdermin D (GSDMD), a key execution protein in NLRP3-driven pyroptosis. Pharmacological inhibition of GSDMD has been shown to block inflammatory cell death while preserving host defense mechanisms (143). Furthermore, physiological interventions such as endurance training have been reported to improve mitochondrial quality control, reduce mitochondrial DAMP release, and attenuate NLRP3 inflammasome activation, suggesting that lifestyle interventions may serve as adjunctive strategies (144).

#### 2.8.2 Clinical-stage strategies

Compared with the extensive preclinical research, clinical-stage interventions targeting the DAMP-NLRP3 axis in COPD/AECOPD remain limited, with most progress derived from autoinflammatory diseases and other chronic inflammatory conditions (145). Dapansutrile (OLT1177), an oral NLRP3 inhibitor, has entered clinical trials for diseases associated with low-grade chronic inflammation, including gout and osteoarthritis. Preclinical studies have demonstrated its efficacy in ameliorating allergic asthma in mice (146), and its favorable safety profile supports further investigation in pulmonary diseases. Notably, first-generation inhibitors such as MCC950 have not advanced to late-stage clinical trials in COPD due to challenges related to formulation, safety, and patient stratification (125).The identification of naturally occurring and disease-associated NLRP3 variants resistant to first-generation inhibitors has driven the development of next-generation compounds. For example, ZAP-180013 has been shown to maintain efficacy against MCC950-resistant variants (147), offering a promising direction to overcome drug resistance. Additionally, biologics targeting upstream inflammatory pathways—such as IL-1 receptor antagonists—have been explored in inflammatory diseases and may offer opportunities for drug repurposing in biomarker-selected AECOPD subgroups (148).

#### 2.8.3 Key translational hurdles: specificity, safety, and patient stratification

Despite progress, translating DAMP-NLRP3-targeting strategies into treatments for AECOPD faces several challenges common to both preclinical and clinical stages. The persistent inflammatory microenvironment in COPD may maintain NLRP3 in a chronically primed state, which could alter its responsiveness to inhibitors, differing from the situation in acute inflammation models (149). Preclinical data derived from acute exposure models may not fully reflect the chronic airway environment of COPD patients, highlighting the need for more clinically relevant animal models. Because NLRP3 serves as a core innate immune sensor, long-term inhibition may increase infection risk, particularly in COPD patients whose airway defense mechanisms are already compromised (150). This concern suggests the need for local administration strategies (e.g., inhaled formulations) to minimize systemic immunosuppression. AECOPD encompasses multiple intrinsic phenotypes driven by infection-triggered, sterile inflammatory, or mixed mechanisms. Reliable stratification biomarkers to identify patients who might benefit from NLRP3-targeted therapy (such as sputum IL-1β levels, caspase-1 activity, or specific DAMP profiles) are still lacking (135). Future clinical trial designs must incorporate biomarker-guided enrollment strategies to improve treatment precision.

Finally, the existence of naturally occurring and disease-associated NLRP3 variants conferring drug resistance constitutes a major obstacle in clinical development (151, 152). Additionally, compensatory activation of alternative inflammasomes following NLRP3 inhibition may limit therapeutic efficacy and requires further investigation. Therefore, future translational research should prioritize the development of patient stratification strategies, explore local administration routes to minimize systemic immunosuppression, and conduct well-designed, biomarker-guided early-phase clinical trials.

### 2.9 Comprehensive analysis of aseptic AECOPD from an immunological perspective

#### 2.9.1 The central role of DAMPs in sterile inflammation

As endogenous danger signals, DAMPs are released from damaged or dying cells and serve to alert the immune system to potential tissue injury. This release can occur due to various stressors, including mechanical injury, ischemia, or cellular stress, leading to a disruption of tissue homeostasis (51). The recognition of DAMPs by innate immune receptors, such as Toll-like receptors and RAGE, triggers a cascade of inflammatory responses designed to restore tissue integrity. However, this response is a double-edged sword; while it is essential for initiating repair processes, excessive or prolonged DAMP signaling can lead to chronic inflammation and tissue damage, exacerbating conditions like COPD (153, 154).

The levels and activity of DAMPs are critical determinants of the intensity and duration of the inflammatory response. For instance, in sterile inflammation, the accumulation of DAMPs can enhance the recruitment of immune cells to the site of injury, promoting a robust inflammatory response. However, the sustained presence of these molecules can lead to a pathological state where inflammation becomes self-perpetuating, contributing to the progression of diseases such as COPD (155). In this context, DAMPs not only signal the presence of injury but also modulate the immune environment, influencing the balance between pro-inflammatory and anti-inflammatory responses. This modulation is particularly evident in macrophage polarization, where DAMPs can dictate whether macrophages adopt a pro-inflammatory M1 phenotype or a pro-resolving M2 phenotype, thus affecting the overall outcome of the inflammatory response.

Moreover, the interplay between DAMPs and other inflammatory mediators is crucial in determining the clinical outcomes of sterile inflammation. For example, the interaction of DAMPs with neutrophils can lead to the formation of neutrophil extracellular traps (NETs), which are implicated in amplifying the inflammatory response (156). This amplification can be particularly detrimental in chronic conditions, where the continuous release of DAMPs from necrotic or stressed cells perpetuates a cycle of inflammation and tissue damage. Therefore, understanding the dynamics of DAMP release and their effects on immune cell behavior is essential for developing therapeutic strategies aimed at mitigating sterile inflammation in diseases like COPD (157).

The fundamental definition of “aseptic” AECOPD demands rigorous re-evaluation. Present clinical protocols classify these events based on the failure to isolate pathogens through conventional culture or PCR; however, such methodologies possess recognized constraints. Routine sputum culturing frequently overlooks fastidious microbes, including *Mycoplasma* and *Chlamydophila*, whereas standard PCR panels often omit emerging viral strains or bacteriophages capable of influencing bacterial virulence. Furthermore, infections characterized by low biomass—such as the intracellular persistence of *Haemophilus influenzae* within epithelial layers—may remain entirely undetected. Consequently, cases designated as “aseptic” may more accurately reflect AECOPD driven by unidentified infectious agents. This diagnostic uncertainty complicates both epidemiological assessments and mechanistic investigations. Should a substantial proportion of “aseptic” episodes be misclassified, current estimates regarding DAMP-mediated sterile inflammation are likely inflated. Future research must leverage metagenomic sequencing alongside host-response classifiers, specifically interferon-stimulated gene signatures, to rigorously rule out occult infection prior to attributing exacerbations to sterile triggers.

#### 2.9.2 The role of NLRP3 inflammasome as an immunological hub

The NLRP3 inflammasome is activated by DAMPs and PAMPs that signal cellular stress or damage. Following activation, NLRP3 drives caspase-1-dependent maturation and secretion of pivotal inflammatory cytokines, including IL-1β and IL-18. Concurrently, NLRP3 activation triggers pyroptosis that further amplifies cytokine release from affected cells, thereby reinforcing the inflammatory milieu (158). By integrating signals from diverse sources such as environmental stressors and endogenous damage, NLRP3 functions as a central regulatory node within the innate immune system, bridging the detection of cellular perturbations to the orchestration of inflammatory responses required for pathogen elimination and tissue restoration.

Furthermore, the NLRP3 inflammasome critically regulates immune response equilibrium, influencing disease progression across various inflammatory disorders. Dysregulated NLRP3 activity—marked by excessive inflammasome activation—is implicated in pathological states such as COPD, where it drives persistent inflammation and tissue injury. This underscores the dual role of NLRP3: while essential for initiating protective immunity, its overactivation can shift responses toward pathology (159). Maintaining a balance between effective immune defense and inflammatory containment is therefore vital, not only for resolving acute infections but also for modulating chronic inflammatory conditions. In the context of sterile inflammation associated with diseases like COPD, DAMP-triggered NLRP3 activation can intensify tissue damage and precipitate clinical exacerbations (91).

Moreover, emerging evidence further reveals that NLRP3 interacts with diverse cellular pathways, including those governing metabolism and autophagy, thereby modulating broader immune responses (160). This crosstalk underscores the functional complexity of NLRP3 beyond cytokine release, situating it within a sophisticated regulatory network that integrates metabolic cues, cellular stress signals, and immune activation to finely calibrate inflammation. Such mechanisms elucidate how dysregulation of NLRP3 can transform normally homeostatic processes into drivers of chronic inflammation. Advancing research into NLRP3 regulatory mechanisms remains essential for developing precise therapies aimed at modulating its activity, particularly in sterile inflammatory diseases such as COPD.

#### 2.9.3 Pathogen and sterile factors’ synergistic pro-inflammatory effects

The NLRP3 inflammasome is activated not only by PAMPs but also by various DAMPs, including ATP and uric acid, which can be released during cellular stress or necrosis (161). The activation of inflammasomes leads to the maturation of pro-inflammatory cytokines such as IL-1β and IL-18, which exacerbate the inflammatory response and contribute to tissue damage (162). This dual activation pathway underscores the complexity of the immune response during acute exacerbations of diseases like COPD, where both infectious and sterile triggers can coexist, leading to a compounded inflammatory state. The intricate immune network that governs the response to these stimuli is shaped by various factors, including the type of cells involved, the nature of the inflammatory signals, and the underlying genetic predispositions of the individual. For example, *in vitro* studies have shown that macrophages play a central role in mediating the inflammatory response by integrating signals from both PAMPs and DAMPs. Upon activation, these immune cells can undergo metabolic reprogramming that enhances their inflammatory capabilities, a process often referred to as “trained immunity.” This phenomenon is characterized by epigenetic changes that prime macrophages for a heightened response to subsequent stimuli, regardless of whether they are pathogenic or sterile (126). The complexity of this immune response can lead to diverse clinical manifestations during AECOPD, ranging from mild symptoms to severe exacerbations that may require hospitalization.

In addition, the diversity of clinical presentations during AECOPD can also be attributed to the differential activation of various immune pathways, influenced by both genetic and environmental factors. For instance, individuals with a history of smoking or those exposed to environmental pollutants may exhibit a more pronounced inflammatory response due to pre-existing lung damage and altered immune function (163). This variability highlights the need for personalized approaches in managing acute exacerbations, taking into consideration the unique interplay of PAMPs and DAMPs in each patient. Furthermore, emerging research suggests that targeting specific components of the inflammasome pathway could provide novel therapeutic avenues for mitigating excessive inflammation and improving clinical outcomes in patients experiencing AECOPD (164).

Although the participation of immune cells is widely acknowledged, the causal trajectory remains ambiguous. It is unclear whether the release of DAMPs initiates immune activation, or if inherent immune defects—such as impaired macrophage-mediated efferocytosis—precipitate secondary necrosis and subsequent DAMP leakage. Current evidence substantiates both hypotheses, indicating a self-perpetuating feedback loop rather than a unidirectional cascade. Nevertheless, the majority of human datasets are cross-sectional, providing merely a static glimpse into a dynamic process. Consequently, longitudinal investigations monitoring immune phenotypes prior to, throughout, and following aseptic exacerbations remain notably limited.

#### 2.9.4 The importance of immune regulatory strategies in clinical management

Recent studies have highlighted the importance of recognizing DAMPs and their contribution to inflammation and immune responses in COPD. For instance, the activation of the NLRP3 inflammasome, a key component of the innate immune response, has been shown to be influenced by DAMPs, leading to the release of pro-inflammatory cytokines such as IL-1β and IL-18, which exacerbate lung inflammation and tissue damage (12). By identifying these sterile inflammatory components, clinicians can optimize treatment strategies that target the specific pathways involved in COPD exacerbations, potentially leading to improved patient outcomes. This tailored approach to treatment can include the use of anti-inflammatory medications, bronchodilators, and novel therapeutic agents that specifically inhibit the activation of inflammasomes or modulate the immune response, thereby reducing the frequency and severity of exacerbations (165).

Moreover, the exploration of immune-targeted therapies presents a promising avenue for reducing the recurrence of exacerbations and improving the overall prognosis for COPD patients. The role of microRNAs, particularly miR-223, has emerged as a significant factor in the regulation of immune responses in COPD. Preclinical studies indicate that miR-223 is involved in the differentiation and activation of immune cells, including monocytes and macrophages, which are critical in the inflammatory processes of COPD (166). Targeting miR-223 and other similar regulatory molecules could provide a novel therapeutic strategy to modulate the immune response, thereby enhancing the effectiveness of existing treatments and potentially leading to a reduction in exacerbation rates. Furthermore, the integration of traditional Chinese medicine (TCM) approaches, such as the use of Bufei Decoction, has shown promise in regulating immune responses and reducing inflammation in preclinical animal models of COPD, suggesting that a multi-faceted approach to treatment may yield better clinical outcomes (167).

The enthusiasm for targeting DAMP/NLRP3 pathways requires tempering in light of three critical challenges. First, as previously highlighted, numerous cases classified as “aseptic” may conceal unidentified pathogens; consequently, anti-inflammatory interventions could unintentionally compromise host defense mechanisms during occult infections. Second, persistent DAMP signaling often triggers compensatory mechanisms, such as the upregulation of alternative inflammasomes or TNF-mediated inflammatory cascades, which may diminish the long-term efficacy of single-agent inhibition. Third, and most importantly, no clinical trial to date has prospectively recruited participants based on verified aseptic conditions combined with elevated DAMP or inflammasome biomarkers. In the absence of such patient enrichment strategies, studies risk obscuring therapeutic benefits within heterogeneous populations—a limitation that undermined previous investigations of broad-spectrum anti-inflammatories in unselected COPD cohorts. Moving forward, progress necessitates precise phenotypic characterization, robust assays to quantify DAMP activity, and adaptive trial frameworks designed to evaluate mechanism-driven hypotheses rather than empirical drug responses.

#### 2.9.5 Promoting the integration of basic and clinical research

Integrated basic and clinical research is essential to advance the understanding and treatment of complex diseases like COPD. Multidisciplinary collaboration facilitates the elucidation of pathophysiological mechanisms and the development of novel therapeutics. For example, the interaction between DAMPs and PAMPs in COPD exacerbations underscores the need for a combined immunological, molecular, and clinical perspective (168). By integrating diverse expertise, researchers can clarify the contribution of inflammasomes such as NLRP3 to dysregulated immune responses in COPD, informing the design of targeted therapies aimed at mitigating inflammation and tissue injury. Preclinical studies demonstrating that NLRP3 inhibition reduces inflammation and attenuates tissue damage in experimental COPD models support the translational potential of such approaches. Moreover, the development of novel interventions—including small-molecule inhibitors or monoclonal antibodies directed against specific inflammatory pathways—requires coordinated efforts between basic and clinical scientists to optimize trial design, patient stratification, and therapeutic application (124).

In addition to fostering drug development, the integration of basic and clinical research promotes the realization of personalized and precision medicine. The heterogeneity observed in COPD patients, characterized by varying responses to treatments and differing disease trajectories, underscores the importance of tailoring therapeutic approaches to individual patient profiles. By utilizing advanced omics technologies—such as genomics, proteomics, and metabolomics—researchers can identify biomarkers that predict treatment responses and disease progression (169). For example, the identification of specific DAMPs that correlate with exacerbation severity in COPD patients could facilitate the stratification of patients based on their inflammatory profiles, allowing clinicians to implement more targeted therapeutic strategies (170). This shift towards individualized treatment plans not only enhances the efficacy of interventions but also minimizes the risk of adverse effects associated with broad-spectrum therapies. Moreover, the integration of clinical data with laboratory findings can inform the development of novel biomarkers that serve as indicators of treatment response, thus enabling real-time monitoring of therapeutic efficacy and facilitating timely adjustments to treatment regimens (171).

The collaboration between basic and clinical researchers is further enhanced by the adoption of innovative research methodologies, including systems biology and computational modeling. These approaches allow for the integration of vast datasets from various sources, providing a holistic view of disease mechanisms and facilitating the identification of potential therapeutic targets. For instance, the application of network analysis to understand the signaling pathways involved in COPD exacerbations can reveal critical nodes that may be amenable to therapeutic intervention (172). Additionally, the use of artificial intelligence and machine learning algorithms in analyzing clinical and biological data can uncover complex relationships that may not be apparent through traditional analytical methods. This convergence of disciplines not only accelerates the pace of discovery but also enhances the translational potential of research findings, ultimately bridging the gap between laboratory and bedside (173).

**Figure 4:**
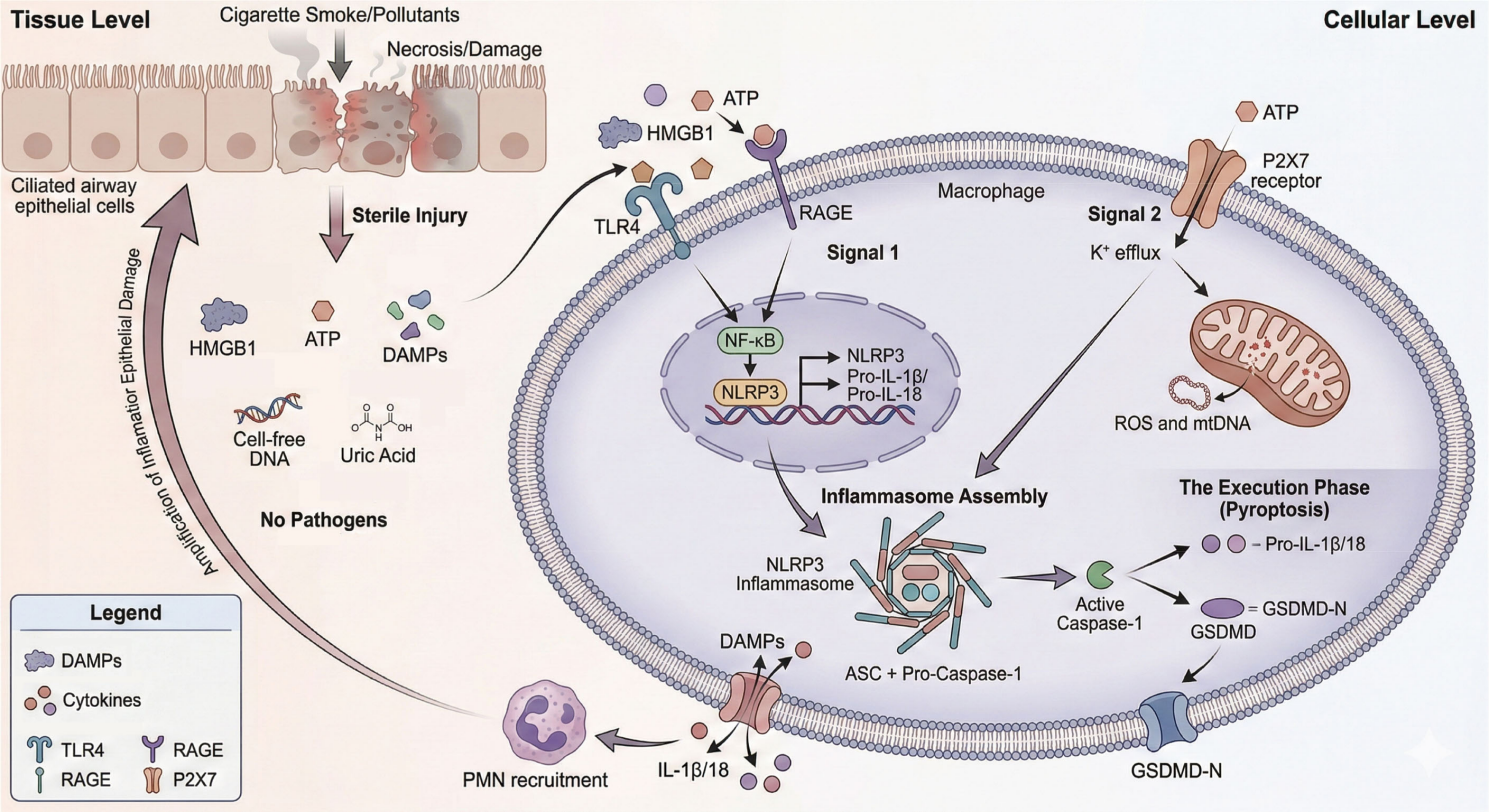
An integrated mechanistic model of sterile inflammation in AECOPD.

An integrated mechanistic model summarizing the interplay among DAMPs, PRRs, NLRP3 inflammasome activation, cytokine cascades, and epithelial injury in sterile AECOPD is presented in **Figure 4**.

### 2.10 Future directions: charting the course for DAMP and inflammasome research in AECOPD

The preceding sections have established the central role of the DAMP-NLRP3 inflammasome axis in the immunopathology of sterile AECOPD. While significant progress has been made, critical knowledge gaps remain that must be addressed to translate these findings into tangible clinical benefits. Future research should focus on the following interconnected priorities:

#### 2.10.1 Deconvoluting the molecular and cellular heterogeneity of DAMP signaling

The response to DAMPs is not uniform but is exquisitely context- and cell-type dependent, necessitating a shift from bulk analysis to single-cell resolution approaches. Future research should prioritize investigating the spatiotemporal dynamics of key DAMPs (e.g., eHsp70, ATP, HMGB1, mitochondrial DNA) within the lung microenvironment during exacerbations using advanced imaging and single-cell RNA sequencing to distinguish driver DAMPs from bystanders (174). Concurrently, efforts must define the cell-type-specific receptor repertoires and signaling pathways engaged by these DAMPs in different pulmonary cells, including how epigenetic modifications such as trained immunity shape these responses. Additionally, exploring the largely uncharted role of other damage-associated molecules like oxidized phospholipids and bioactive lipids in NLRP3 inflammasome activation represents a promising frontier with high therapeutic potential (175).

#### 2.10.2 The functional dichotomy of DAMPs: implications for precision medicine

The patient population with AECOPD is highly heterogeneous, and emerging evidence suggests that the spectrum of DAMPs that trigger or sustain inflammation varies significantly among individuals and exacerbation phenotypes. For instance, an exacerbation dominated by the S100A8/A9-RAGE axis likely involves prominent sterile inflammation resulting from potent activation of the NLRP3 inflammasome (66, 67). Conversely, infectious exacerbations primarily triggered by PAMPs may involve a distinct combination of signaling pathways. Furthermore, DAMPs such as mtDNA, released due to chronic mitochondrial dysfunction, may not act as acute triggers but rather as chronic drivers of inflammation and tissue remodeling, potentially contributing to long-term disease progression and increased mortality risk (71, 72).

This functional dichotomy among DAMPs calls for a paradigm shift in precision medicine strategies in AECOPD management. Future therapeutic approaches may require patient stratification based on biomarker profiles, such as specific DAMP levels (e.g., S100A8/A9, HMGB1) or signatures indicating activation of inflammatory pathways (e.g., NLRP3/caspase-1 activity). Accordingly, interventions targeting “acute trigger” DAMPs—such as neutralizing antibodies against eHsp70 or S100A8/A9, or antagonists of their receptors (e.g., TLR4, RAGE)—may be optimally employed to prevent or treat specific types of non-infective exacerbations. Conversely, modulating pathways associated with “chronic driver” DAMPs, such as inhibitors of the cGAS-STING axis—which is activated by dsDNA/mtDNA—to dampen sustained interferon and inflammatory responses, might be more suitable for mitigating long-term disease progression and remodeling. The exploration of such targeted strategies, including the inhibition of specific inflammasomes like NLRP3, represents a promising frontier for innovative therapies. However, a major translational bottleneck remains the current lack of rapid, reliable, and clinically validated point-of-care biomarkers capable of accurately defining these DAMP-driven endotypes in real-time. Such biomarkers are crucial for guiding targeted therapeutic decisions.

#### 2.10.3 Targeting the NLRP3 inflammasome: towards a new generation of therapeutics

While inhibitors like MCC950 have validated NLRP3 as a therapeutic target, their clinical translation faces hurdles related to specificity, bioavailability, and long-term safety, prompting the need for next-generation approaches. Future therapeutic development should focus on creating highly selective, orally bioavailable small molecule inhibitors that target distinct activation steps or specific protein-protein interactions (e.g., NEK7-NLRP3) to minimize off-target effects, while also exploring natural compounds with potent NLRP3 inhibitory activity (176). Beyond direct NLRP3 inhibition, strategies should include assessing the therapeutic potential of modulating upstream DAMP receptors (e.g., P2X7 antagonists, RAGE inhibitors) and conducting clinical trials with existing IL-1 receptor antagonists (e.g., anakinra) in well-defined AECOPD patient subsets (177). Furthermore, investigating therapies that specifically block the pyroptotic cell death pathway (e.g., gasdermin D inhibitors) could preserve host defense mechanisms while preventing the tissue destruction caused by lytic cell death (178). Critically, advancing these therapies requires robust clinical trials designed to evaluate not only clinical outcomes but also the underlying immunological changes with treatment. Collaborative efforts between basic scientists and clinical researchers are essential to ensure preclinical insights are effectively translated into practice, ultimately reducing the burden of AECOPD (179).

## 3 Conclusion

COPD exacerbations represent a significant clinical challenge due to their complex immunopathological underpinnings. This review synthesizes these complexities into an integrated conceptual framework, unifying DAMPs, PRRs, NLRP3 inflammasome activation, cytokine cascades, and epithelial damage into a coherent pathogenic model. Sterile AECOPD is initiated by the release of “acute trigger” DAMPs, such as S100A8/A9 and ATP, from stressed airway epithelial cells, which activate resident immune cells via RAGE, TLR4, and P2X7 receptors to provide Signal 1 (priming) for NLRP3 inflammasome activation. Subsequently, factors including potassium efflux, mitochondrial dysfunction, and reactive oxygen species provide Signal 2, triggering NLRP3 assembly and caspase-1 activation. The activated caspase-1 processes IL-1β and IL-18, driving a cytokine storm, and cleaves gasdermin D to induce pyroptosis. Pyroptosis leads to the release of a second wave of intracellular DAMPs, creating a self-amplifying cycle of epithelial injury, inflammasome activation, and further inflammation. Within this cycle, “chronic burden” DAMPs such as mtDNA contribute to the baseline inflammatory milieu, while viral infections synergistically amplify the cascade by providing both PAMPs and cellular stress signals. From an expert perspective, understanding this intricate interplay—centered on DAMP heterogeneity, NLRP3 inflammasome as a signaling hub, pyroptosis as an amplification mechanism, and viral synergy as a modifying factor—is essential for advancing both the scientific knowledge and therapeutic management of COPD exacerbations.

COPD exacerbations represent a significant clinical challenge due to their complex immunopathological underpinnings. This review has highlighted the pivotal role of DAMPs, particularly eHsp70 and ATP, orchestrate sterile inflammation through activation of the NLRP3 inflammasome. From an expert perspective, understanding this intricate interplay is essential for advancing both the scientific knowledge and therapeutic management of COPD exacerbations.

The evidence underscores that DAMPs function not only as pro-inflammatory signals but also as critical modulators of inflammasome activity, thereby shaping the local airway immune milieu. This dual role complicates the inflammatory cascade, as DAMPs can amplify immune responses while simultaneously influencing regulatory pathways that determine the magnitude and duration of inflammation. Moreover, viral infections, such as the influenza virus, exacerbate this scenario by potentiating NLRP3 inflammasome activation, which accelerates inflammatory deterioration during acute exacerbations. This synergy between pathogen-induced and sterile inflammation highlights the necessity of a nuanced approach in dissecting COPD exacerbation mechanisms.

Balancing these diverse research findings reveals a landscape where the NLRP3 inflammasome emerges as a central node integrating multiple inflammatory signals. Therapeutic strategies targeting the NLRP3 inflammasome and its regulatory network have shown promising preclinical and early clinical results. Such interventions hold the potential to mitigate excessive inflammation, preserve lung function, and ultimately improve patient outcomes. However, the heterogeneity of COPD pathophysiology and the dynamic nature of immune responses necessitate precision in targeting these pathways to avoid unintended immunosuppression or inadequate efficacy.

Future research must therefore prioritize elucidating the precise molecular mechanisms governing DAMP-mediated NLRP3 inflammasome activation in the COPD airway environment. This includes characterizing the temporal and spatial dynamics of inflammasome assembly, identifying key regulatory checkpoints, and understanding patient-specific variations influenced by genetic and environmental factors. Integrating multi-omics approaches and advanced imaging techniques could accelerate these insights, enabling the development of biomarkers for patient stratification and treatment monitoring.

Yet a fundamental constraint on such efforts lies in the current evidence base itself. The majority of mechanistic data supporting the DAMP–NLRP3 axis in AECOPD originate from preclinical models that capture only a fraction of human disease pathophysiology. Standard murine protocols—employing acute cigarette smoke exposure, elastase challenge, or sterile particulate instillation—generate transient, synchronized inflammation but fail to recapitulate the chronicity, systemic comorbidities, and polypharmacy that define clinical COPD. Critically, sterile exacerbations in patients typically arise against a backdrop of persistent low-grade DAMP release from senescent or metabolically stressed cells, a scenario rarely modeled in animals. As a result, therapeutic responses observed in mice, such as the potent suppression of inflammation by MCC950, may not reliably predict outcomes in heterogeneous human populations lacking biomarker-guided selection. Moreover, direct longitudinal evidence linking specific DAMP–inflammasome interactions to clinical trajectories in rigorously phenotyped sterile AECOPD cohorts remains scarce, with most human studies limited to cross-sectional designs or mixed exacerbation etiologies. Bridging this translational gap necessitates prospective studies with serial sampling across the full exacerbation cycle—from stability through acute worsening to recovery—in well-defined patient subgroups.

While the pathogenic role of the DAMP-NLRP3 axis in AECOPD is well-substantiated, the existing evidence remains incomplete. Insights gleaned from research on other chronic conditions—such as neurodegenerative, autoimmune, and metabolic diseases—have significantly advanced our understanding of this pathway. However, these studies also highlight the critical importance of tissue-specific differences and differences in disease context. Therefore, when extrapolating these findings to AECOPD, it is imperative to critically assess their relevance. Future investigations should focus on several key directions. First, dynamic quantification of the release of specific DAMPs, as well as establishing their causal relationship with NLRP3 activation in models that better mimic the clinical AECOPD scenario (e.g., viral infection superimposed on elastase injury), is needed. Second, employing techniques such as single-cell sequencing to delineate the specific cell populations within the AECOPD airway (e.g., macrophage subsets, airway epithelial cells) that are the primary contributors to NLRP3 activation will provide crucial cellular resolution. Third, conducting exploratory clinical trials to evaluate the efficacy and safety of NLRP3 inhibitors in stratified patient populations using clear biomarkers (e.g., sputum IL-1β, caspase-1 activity) is essential for clinical translation. Together, these efforts will advance the field from descriptive associations to mechanism-based interventions.

Furthermore, translating these mechanistic insights into clinical practice demands robust clinical trials designed to evaluate targeted immunomodulatory therapies in well-defined COPD subpopulations. Emphasizing individualized treatment paradigms will be critical, as the immunopathology of COPD exacerbations varies widely among patients. Incorporating real-world data and adaptive trial designs may facilitate this precision medicine approach, ultimately leading to more effective and safer interventions.

In conclusion, the complex immunopathogenesis of AECOPD, driven by DAMP-induced NLRP3 inflammasome activation and modulated by viral co-factors, presents both challenges and opportunities. From an expert sta()ndpoint, harmonizing diverse research perspectives underscores the inflammasome as a promising therapeutic target. Advancing our understanding of its regulation and clinical translation holds the key to developing personalized immunotherapies that can transform the management of COPD exacerbations and improve patient quality of life.
